# Polyhydroxy *p*-Terphenyls from a Mangrove Endophytic Fungus *Aspergillus candidus* LDJ-5

**DOI:** 10.3390/md19020082

**Published:** 2021-02-02

**Authors:** Guoliang Zhou, Xiaomin Zhang, Mudassir Shah, Qian Che, Guojian Zhang, Qianqun Gu, Tianjiao Zhu, Dehai Li

**Affiliations:** 1 Key Laboratory of Marine Drugs, Chinese Ministry of Education, School of Medicine and Pharmacy, Ocean University of China, Qingdao 266003, China; zhouguoliang@ouc.edu.cn (G.Z.); zhangxiaomin@ouc.edu.cn (X.Z.); s84mudassir@gmail.com (M.S.); cheqian064@ouc.edu.cn (Q.C.); zhangguojian@ouc.edu.cn (G.Z.); guqianq@ouc.edu.cn (Q.G.); 2 Laboratory for Marine Drugs and Bioproducts, Pilot National Laboratory for Marine Science and Technology, Qingdao 266237, China; 3 Open Studio for Druggability Research of Marine Natural Products, Pilot National Laboratory for Marine Science and Technology, Qingdao 266237, China

**Keywords:** *Aspergillus candidus*, *p*-terphenyl dimer, anti-influenza virus A (H1N1) activity, protein tyrosine phosphatase 1B (PTP1B) inhibitory activity

## Abstract

Six undescribed polyhydroxy *p*-terphenyls, namely asperterphenyllins A–F, were isolated from an endophytic fungus *Aspergillus candidus* LDJ-5. Their structures were determined by NMR and MS data. Differing from the previously reported *p*-terphenyls, asperterphenyllin A represents the first *p*-terphenyl dimer connected by a C-C bond. Asperterphenyllin A displayed anti-influenza virus A (H1N1) activity and protein tyrosine phosphatase 1B (PTP1B) inhibitory activity with IC_50_ values of 53 μM and 21 μM, respectively. The anti-influenza virus A (H1N1) activity and protein tyrosine phosphatase 1B (PTP1B) inhibitory activity of *p*-terphenyls are reported for the first time. Asperterphenyllin G exhibited cytotoxicity against nine cell lines with IC_50_ values ranging from 0.4 to 1.7 μM. Asperterphenyllin C showed antimicrobial activity against *Proteus* species with a MIC value of 19 μg/mL.

## 1. Introduction

*p*-Terphenyls are aromatic hydrocarbons consisting of a chain of three benzene rings. Most *p*-terphenyls have been isolated from macrofungi, such as *Paxillus curtisii*, *P. atrotomentosus*, *Thelephora aurantiotincta*, and *T. ganbajun*. A few examples have also been reported from endolichenic fungi, actinomycetes, and mosses [1,2]. *Aspergillus candidus* has been identified as one of the main deterioration fungi in the storage of grain [3]. So far, a variety of secondary metabolites have been reported from *A. candidus*, such as terpenoids, flavones, and indoles [3,4,5]. Additionally, over 20 *p*-terphenyls have been isolated from *A. candidus* [2,3,6,7]. The structural diversity of *p*-terphenyls is mainly due to the substitution on ring B and the connections with other rings, for example, *p*-terphenyls bearing three or four oxygenated functions at the central ring [6,7,8,9,10], benzofuranoid *p*-terphenyls [10,11,12], *p*-terphenyls with a *para* quinone function at the central ring [13,14], nitrogenous-containing *p*-terphenyls [15,16,17], and other *p*-terphenyl derivatives [18,19]. The carbon skeletons usually bear oxygen functions including hydroxy, methoxy, and ester groups, mostly at C-3, C-4, C-3”, or/and C-4” [1,2]. Recently, there has been a growing number of new types of *p*-terphenyls discovered, e.g., the allantonaphthofurans [20] and hawaiienols [21]. *p*-Terphenyls are also attractive because of their broad bioactivities such as cytotoxic, antimicrobial, antioxidant, and *α*-glucosidase inhibitory effects [1,8,22,23]. Among these activities, the cytotoxicity is the most widely studied, and the mechanisms of action of certain *p*-terphenyls have also been elucidated [1,7].

**Figure 1:**
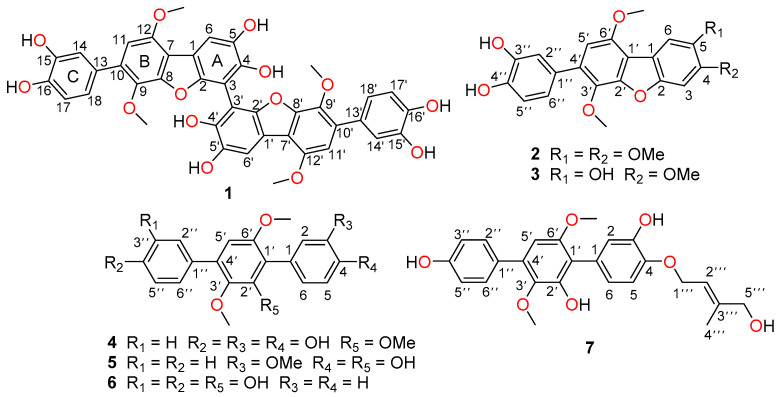
Structures of **1**–**7**.

In our previous research, nine cytotoxic *p*-terphenyls had been discovered from an endophytic fungus *Aspergillus candidus* LDJ-5, isolated from the root of *Rhizophora apiculata* Blume collected from Sanya Bailu Park of Hainan Province, China [6]. Through scale-up of the same fermentation, we further focused on HPLC and LC-MS analysis of fractions containing minor *p*-terphenyl constituents that resulted in the isolation and identification of six undescribed *p*-terphenyls, namely asperterphenyllins A–F (**1**–**6**), and one new naturally occurring product, asperterphenyllin G (**7**) (Figure 1). Herein we report the isolation, structure elucidation, and bioactivities of these compounds.

## 2. Results and Discussion

The fungal strain *Aspergillus candidus* LDJ-5 was fermented (50 L) under static conditions at 28 °C for 30 days. The EtOAc extract (50 g) of the fermentation was fractionated repeatedly by silica gel column chromatography, C-18 column chromatography, Sephadex LH-20 column chromatography, ODS MPLC, and finally HPLC to yield **1**–**7**.

**Figure 2:**
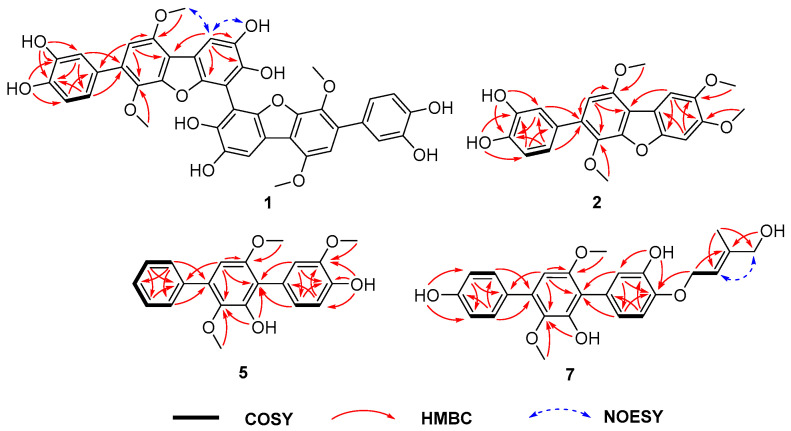
Selected ^1^H-^1^H COSY, HMBC, and NOESY correlations of compounds **1**, **2**, **5** and **7**.

Asperterphenyllin A (**1**) was obtained as a yellow amorphous solid. The molecular formula of **1** was determined as C_40_H_30_O_14_ on the basis of NMR and HRESIMS (Figures S1–S8). Analysis of the ^1^H, ^13^C, and HSQC NMR data of **1** revealed the presence of signals attributable to two methoxy groups (*δ*_C_: 56.3, *δ*_H_: 4.01; *δ*_C_: 60.7, *δ*_H_: 3.51), five protonated sp^2^ carbons (*δ*_C_: 105.9, *δ*_H_: 6.69; 106.6, *δ*_H_: 7.51; 115.9, *δ*_H_: 6.77; 117.2, *δ*_H_: 6.97; 120.7, *δ*_H_: 6.82), and thirteen non-protonated sp^2^ carbons (*δ*_C_: 105.1, 113.2, 114.6, 129.6, 130.9, 136.3, 142.8, 144.7, 145.1, 145.2, 148.7, 148.9, 149.7) (Table 1). The presence of only 20 signals in the ^13^C NMR spectrum, in combination with the molecular formula, indicated that **1** was a symmetrical dimer. The NMR data for compound **1** were highly similar to those of candidusin B [24], and the major differences were the absence of the H-3 signal at *δ*_H_ 7.06 (s) and the higher chemical shift of C-3 (*δ*_C_ + 6.7 ppm), suggesting that the two monomers were connected through C-3. The structure was further confirmed by interpretation of 2D NMR spectra (^1^H-^1^H COSY, HMBC, and NOESY), especially the HMBC correlations from H-6 to C-2, C-4, C-5, and C-7 (Figure 2). Therefore, the structure of compound **1** was determined to be a dimer of candidusin B. Compound **1** contains the biphenyl system (C3-C3′), which could generate atropisomers. Notably, the specific optical rotation value of **1** was zero, suggesting a racemic mixture. Subsequent chiral HPLC analysis of **1** showed that compound **1** was a pair of enantiomers with about a 1:1 ratio (Figure S53). However, it was difficult for the enantiomers to be baseline separated under the chromatographic conditions.

The molecular formula of asperterphenyllins B (**2**) and C (**3**) were determined as C_22_H_20_O_7_ and C_21_H_18_O_7_ by HRESIMS, respectively. The ^1^H and ^13^C NMR spectra of **2** were similar to those of candidusin B except for the presence of two methoxy groups and the absence of the two hydroxy groups at C-4 and C-5 [24]. The positions of the methoxy groups were established by HMBC correlations from the proton signal at *δ*_H_ 3.87 (OMe-4) to the carbon at *δ*_C_ 149.6 (C-4), and from *δ*_H_ 3.86 (OMe-5) to *δ*_C_ 146.6 (C-5) (Figure 2). The difference between **3** and **2** was the replacement of the methoxy group at C-5 in **2** by a hydroxy group in **3**, which was confirmed by the HMBC correlation from OH-5 to C-4, C-5, and C-6.

Asperterphenyllins D (**4**), E (**5**), and F (**6**) were obtained as colorless powders. Their molecular formulae were determined as C_21_H_20_O_6_, C_21_H_20_O_5,_ and C_20_H_18_O_5_ by HRESIMS, respectively. Their ^1^H and ^13^C NMR spectra (Table 2 and Table 3) resembled those of 3′-*O*-methylterphenyllin [9], 4”-deoxyisoterprenin [25], and 3,3”-dihydroxyterphenillin [26], respectively. The difference between **4** and 3′-*O*-methylterphenyllin was the presence in **4** of a hydroxy group at C-3, which was confirmed by the HMBC correlations from H-2 to C-1′, C-4, and C-6, from H-5 to C-1 and C-3, and from H-6 to C-1′, C-2, and C-4, as well as by the chemical shift value of C-3 (*δ*_C_: 145.1). On the other hand, the main difference between compound **5** and 4”-deoxyisoterprenin was the lack of an oxygenated isoprenoid unit at C-3 and the presence of a methoxy group instead. On the other hand, **6** differs from 3,3”-dihydroxyterphenillin by the absence of the two hydroxy groups on ring A.

Compound **7** was previously synthesized by Kenji et al. (1998), and only its ^1^H NMR data were reported [27]. This is the first time it has been isolated from a natural resource. The ^1^H NMR data of compound **7** were in agreement with the data reported, and the structure was also supported by the HREIMS, ^13^C NMR data, COSY, and HMBC correlations (Figure 2). Since this is the first isolation of **7** from a natural source, it was named asperterphenyllin G.

Generally, *p*-terphenyls with a 1,2,4-trisubstituted ring B and benzofuranoid *p*-terphenyls are not axially chiral [6,8,9]. Additionally, no optical rotations were observed for compounds **2**–**7**. Thus, compounds **2**–**7** do not have axial chirality.

Although new members of *p*-terphenyls are constantly being disclosed, to our best knowledge, asperterphenyllin A (**1**) represents the first *p*-terphenyl dimer connected through a C-C bond. The naturally occurring *p*-terphenyls with the hydroxy group at C-5‴ on the prenyl chain (such as **7**) are rare, with only three cases previously reported (arenarins B and C and prenylterphenyllin F) [6,28].

All *p*-terphenyls (**1**–**7**) were tested for their cytotoxicity against the L-02, MGC-803, HCT-116, BEL-7402, A549, SH-SY5Y, Hela, U87, K562, HL-60, HO8910, and MCF-7 cell lines using either the SRB or the MTT method with adriamycin as positive control. Compound **7** exhibited broad activities against the L-02, MGC-803, HCT-116, BEL-7402, A549, SH-SY5Y, Hela, U87, and HO8910 cell lines with IC_50_ values of 1.7, 1.0, 0.8, 6.0, 0.4, 0.6, 1.7, 0.9, and 1.3 μM, respectively, while compounds **1**–**6** exhibited IC_50_ > 50 μM against all tested cell lines. Recently, we have also reported nine prenylated *p*-terphenyls from *Aspergillus candidus* LDJ-5 [6]. Most of the prenylated *p*-terphenyls were cytotoxic. Additionally, comparing to prenylcandidusin B and prenylcandidusin C, compounds **2** and **3** were distinguished by the presence of one hydroxy group and the lack of the isoprenyl group on ring C (C-3″). Prenylcandidusin B and prenylcandidusin C were reported to show moderate inhibitory activity against K562 cell lines [3], while compounds **2** and **3** did not show any cytotoxicity effects. These results suggest that the presence of isoprenyl or *O*-isoprenyl groups in *p*-terphenyls might play a key role in cytotoxicity effects. The antimicrobial activities of compounds **1**–**7** were evaluated in vitro against *Proteus* species, *Pseudomonas aeruginosa*, *Bacillus subtilis*, *B. cereus*, and *Mycobacterium phlei*. Compound **3** showed the best activity against *Proteus* species with a MIC value of 19 μg/mL (Table S1). Asperterphenyllin A (**1**) was also tested for antiviral activity and protein tyrosine phosphatase 1B (PTP1B) inhibitory activity. The antiviral activity of asperterphenyllin A (**1**) was evaluated against the influenza A virus (H1N1) using the cytopathic effect (CPE) inhibition assay. Compound **1** exhibited inhibitory effects with an IC_50_ value of 53 μM (ribavirin as a positive control, IC_50_ = 35 μM). Protein tyrosine phosphatase 1B (PTP1B) has been reported to be a novel drug target for diabetes and obesity. It has also been considered to be involved in tumorigenesis [29,30]. Compound **1** was tested for its inhibitory activity against protein tyrosine phosphatase 1B (PTP1B), and showed inhibitory activity with an IC_50_ value of 21 μM.

## 3. Materials and Methods

### 3.1. General Experimental Procedures

Specific rotations were obtained on a JASCO P-1020 digital polarimeter developed by JASCO Corporation, Tokyo, Japan. UV spectra were carried out on Waters 2487 developed by Waters Corporation, Milford, MA, USA. NMR spectra were recorded on Agilent 500 MHz DD2 spectrometers made by Agilent Technologies Inc., Santa Clara, CA, USA, using tetramethylsilane as an internal standard, and the chemical shifts were recorded in *δ* values. HRESIMS spectra were obtained on a LTQ Orbitrap XL mass spectrometer made by Thermo Fisher Scientific, Waltham, MA, USA. The compounds were purified by HPLC made by the Waters company, Milford, MA, USA, equipped with a 2998 PDA detector and a C18 column (YMC-Pack ODS-A, 10 × 250 mm, 5 µm, 3 mL/min). Medium-pressure preparative liquid chromatography (MPLC) was performed on a Bona-Agela CHEETAH HP100 made by Beijing Agela Technologies Co., Ltd., Beijing, China. Column chromatography (CC) was performed with silica gel (100−200 mesh, 200−300 mesh, Qingdao Marine Chemical Inc, Qingdao, China) and Sephadex LH-20 (Amersham Biosciences, San Francisco, CA, USA), respectively. LC-MS was recorded in ESI mode on an Acquity UPLC H-Class connected to a SQ Detector 2 mass spectrometer using a BEH C18 column (1.7 µm, 2.1 × 50 mm, 1 mL per minute) constructed by Waters Corporation, Milford, CT, USA.

### 3.2. Fungal Material

The fungus was isolated from the root of *Rhizophora apiculata* Blume in the Sanya Bailu Park of Hainan Province, China. It was identified as *Aspergillus candidus* (GenBank accession number: MK209104) based on ITS sequence. The fungal sample was deposited at the Key Laboratory of Marine Drugs, the Ministry of Education of China, School of Medicine and Pharmacy, Ocean University of China, Qingdao, People’s Republic of China.

### 3.3. Fermentation and Extraction

The fungus was cultured under static conditions in 166 Erlenmeyer flasks (1 L flasks with 300 mL of culture medium per flask comprising 2% mannitol, 1% monosodium glutamate, 3% maltose, 0.3% yeast extract, 1% glucose, 0.1% corn steep liquor, 0.03% magnesium sulfate heptahydrate, 0.05% monopotassium phosphate in fresh water, autoclaved at 121 °C for 20 min before inoculation). After 30 days of cultivation at 28 °C, 50 L of the whole broth was filtered through a cheesecloth to separate the supernatant from the mycelia. The former was extracted three times with EtOAc, while the latter was extracted three times with methanol and concentrated under reduced pressure to afford an aqueous solution, which was extracted three times with EtOAc. Two EtOAc solutions were combined and concentrated under reduced pressure to get the organic extract (50 g). 

### 3.4. Isolation 

The crude extract (50 g) was subjected to a vacuum liquid silica gel column chromatography (VLC) using a gradient solvent system of MeOH-CH_2_Cl_2_ to obtain nine fractions (Fr.1–9). Fr.5 was further applied on a C-18 ODS column using a step gradient elution of MeOH:H_2_O to yield five subfractions (sfr.5.1–5.5). sfr.5.2 eluted with MeOH was fractionated on a MPLC (C-18 ODS) using a gradient solvent system of MeOH-H_2_O (from 30% MeOH to 100% MeOH) to give six sub-subfractions (ssfr.5.2.1–5.2.6). ssfr.5.2.5 was then subjected to a semi-preparative HPLC (MeCN:H_2_O, 24:78, 3 mL/min) to give five fractions (sssfr.5.2.5.1–5.2.5.5). Compound **1** (3.0 mg, *t*_R_ 32.0 min) was obtained from sssfr.5.2.5.5 by semi-preparative HPLC (MeCN:H_2_O, 20:80, 3 mL/min). Compound **4** (3.0 mg, *t*_R_ 20.0 min) was obtained from sssfr.5.2.5.2 by semi-preparative HPLC (MeCN:H_2_O, 21:79, 3 mL/min). Compound **7** (4.6 mg, *t*_R_ 27.5 min) was obtained from sssfr.5.2.5.4 by semi-preparative HPLC (MeCN:H_2_O, 22:78, 3 mL/min). Fr.4 was subjected to a C-18 ODS column using a step gradient elution of MeOH:H_2_O to yield five subfractions (sfr.4.1–4.5). sfr.4.3 was subjected to semi-preparative HPLC (MeCN:H_2_O, 18:82, 3 mL/min) to give three sub-subfractions (ssfr.4.3.1–4.3.3). Compound **2** (5.0 mg, *t*_R_ 17.0 min) was then obtained from ssfr.4.3.1 by semi-preparative HPLC (MeCN:H_2_O, 32:68, 3 mL/min). Compound **5** (2.7 mg, *t*_R_ 23.0 min) was obtained from ssfr.4.3.2 by semi-preparative HPLC (MeCN:H_2_O, 45:55, 3 mL/min). sfr.4.2 was subjected to a C-18 ODS column using a step gradient elution of MeOH:H_2_O to yield six sub-subfractions (ssfr.4.2.1–4.2.6). Compound **3** (4.5 mg, *t*_R_ 12.5 min) was obtained from ssfr.4.2.6 by semi-preparative HPLC (MeCN:H_2_O, 42:58, 3 mL/min). Compound **6** (6.2 mg, *t*_R_ 27.0 min) was obtained from ssfr.4.2.5 by semi-preparative HPLC (MeOH:H_2_O, 33:67, 3 mL/min).

*Asperterphenyllin A* (**1**): yellow, amorphous solid; ${\left[\alpha \right]}_{D}^{20}$ 0 (*c* 1.00, MeOH); UV (MeOH) λ_max_ (log ε): 217 (6.12), 292 (1.25), 338 (2.03) nm; IR (KBr) *ν*_max_ 3377, 2842, 2254, 1682, 1592, 1529, 1450, 1387, 1192, 1099, 1024, 826 cm^−1^; ^1^H and ^13^C NMR see Table 1; HRESIMS *m/z* 733.1543 [M − H]^−^ (calcd. C_40_H_29_O_14_, 733.1563).

*Asperterphenyllin B* (**2**): colorless, amorphous solid; UV (MeOH) λ_max_ (log ε): 219 (4.77), 298 (1.29), 330 (3.31) nm; IR (KBr) *ν*_max_ 3396, 2932, 1683, 1609, 1483, 1439, 1386, 1208, 1133, 1072, 1019, 845 cm^−1^; ^1^H and ^13^C NMR see Table 2; HRESIMS *m/z* 397.1277 [M + H]^+^ (calcd. C_22_H_21_O_7_, 397.1282).

*Asperterphenyllin C* (**3**): colorless, amorphous solid; UV (MeOH) λ_max_ (log ε): 210 (7.05), 297 (1.45), 332 (2.35) nm; IR (KBr) *ν*_max_ 3362, 2933, 2841, 1681, 1592, 1480, 1439, 1394, 1355, 1277, 1240, 1185, 1129, 1099, 1070, 1024, 820 cm^−1^; ^1^H and ^13^C NMR see Table 2; HRESIMS *m/z* 383.1119 [M + H]^+^ (calcd. C_21_H_19_O_7_, 383.1125).

*Asperterphenyllin D* (**4**): colorless, amorphous solid; UV (MeOH) λ_max_ (log ε): 212 (5.15), 290 (2.35) nm; IR (KBr) *ν*_max_ 3399, 2937, 2850, 1683, 1610, 1519, 1457, 1394, 1230, 1174, 1110, 1024, 834 cm^−1^; ^1^H and ^13^C NMR see Table 2; HRESIMS *m/z* 367.1182 [M − H]^−^ (calcd. C_21_H_19_O_6_, 367.1187).

*Asperterphenyllin E* (**5**): colorless, amorphous solid; UV (MeOH) λ_max_ (log ε): 219 (4.88), 271 (1.76) nm; IR (KBr) *ν*_max_ 3355, 2937, 2841, 1683, 1599, 1521, 1483, 1459, 1404, 1360, 1304, 1206, 1119, 1073, 1029, 826 cm^−1^; ^1^H and ^13^C NMR see Table 3; HRESIMS *m/z* 353.1383 [M + H]^+^ (calcd. C_21_H_21_O_5_, 353.1384).

*Asperterphenyllin F* (**6**): colorless, amorphous solid; UV (MeOH) λ_max_ (log ε): 208 (6.28), 281 (2.34) nm; IR (KBr) *ν*_max_ 3414, 2931, 2853, 1682, 1526, 1483, 1441, 1405, 1384, 1208, 1141, 1068, 1027, 840 cm^−1^; ^1^H and ^13^C NMR see Table 3; HRESIMS *m/z* 339.1222 [M + H]^+^ (calcd. C_20_H_19_O_5_, 339.1227).

*Asperterphenyllin G* (**7**): colorless, amorphous solid; UV (MeOH) λ_max_ (log ε): 212 (5.24), 275 (2.12) nm; IR (KBr) *ν*_max_ 3397, 2936, 1681, 1611, 1523, 1489, 1457, 1405, 1384, 1243, 1116, 1072, 1023, 825 cm^−1^; ^1^H and ^13^C NMR see Table 3; HRESIMS *m/z* 439.1750 [M + H]^+^ (calcd. C_25_H_27_O_7_, 439.1751).

### 3.5. Cytotoxicity Assay 

The cytotoxicity of **1**–**7** and positive control was evaluated against human leukemia cell lines K562 and HL-60 (using the MTT method), human normal liver cell line L-02, human gastric cancer cell lines MGC-803, human colon cancer lines HCT-116, human hepatocacinoma cell line BEL-7402, human lung cancer cell lines A549, human neuroblastoma cell line SH-SY5Y, human cervical cancer cell lines HeLa, human glioma cell lines U87, human ovarian cancer cell lines HO-8910, and human breast cancer cell lines MCF-7 (using the SRB method). Adriamycin was used as positive control. The detailed methodologies for biological testing have been described in previous reports [31]. All the cell lines were purchased from the Institute of Biochemistry and Cell Biology, Chinese Academy of Sciences (Shanghai, China).

### 3.6. Anti-Influenza A viral (H1N1) Assay 

The antiviral activity of compound **1** against influenza A virus (H1N1) was evaluated by the CPE inhibition assay. The detailed methodologies for biological testing have been described in our previous report [32].

### 3.7. Antimicrobial Activities 

Antimicrobial activities were evaluated as previously reported by using the agar dilution method [33]. The five microbial strains included *Proteus* sp., *Pseudomonas aeruginosa*, *Bacillus subtilis*, *B. cereus*, and *Mycobacterium phlei*. Ciprofloxacin was used as positive control. All strains were donated by the Qingdao municipal hospital.

### 3.8. PTP1B Inhibitory Assay 

PTP1B activity of compound **1** was measured as the rate of hydrolysis of *p*-nitrophenyl phosphate (*p*NPP) in a 96-well microtiter plate format. Standard assays were conducted at room temperature in a total volume of 0.2 mL that contained CPBS buffer (50 mM), NaCl (100 mM), EDTA (1 mM), DTT (1 mM), *p*NPP (2 mM), and PTP1B (0.3 μg/mL). Ursolic acid was used as the positive control. Inhibitors were added in DMSO at 100 times the final concentration. PTP1B activity was calculated by the cleavage of the *p*NPP and the resulted production of *p*-nitrophenol (*p*NP). The enzyme activity was estimated by measuring the absorbance at 405 nm with appropriate corrections. Each experiment was performed in triplicate, and IC_50_ data were derived from three independent experiments [34].

## 4. Conclusions

In summary, seven compounds, including six undescribed *p*-terphenyls asperterphenyllins A–F (**1**–**6**), and one new natural product asperterphenyllin G (**7**), were isolated from a mangrove-derived fungus *A. candidus* LDJ-5. Asperterphenyllin A (**1**) represents the first *p*-terphenyl dimer connected through a C-C bond, and displayed anti-influenza virus A (H1N1) activity and protein tyrosine phosphatase 1B (PTP1B) inhibitory activity with the IC_50_ values of 53 μM and 21 μM, respectively. To the best of our knowledge, the anti-influenza virus A (H1N1) activity and protein tyrosine phosphatase 1B (PTP1B) inhibitory activity of *p*-terphenyls have not previously been reported. Asperterphenyllin C (**3**) showed antimicrobial activity against *Proteus* species with a MIC value of 19 μg/mL. Asperterphenyllin G (**7**) had a hydroxyprenyl group on ring A and exhibited cytotoxicity against nine cell lines with IC_50_ values ranging from 0.4 to 1.7 μM. 

## Figures and Tables

**Table 1:** ^1^H (500 MHz) and ^13^C NMR (125 MHz) data of **1** in DMSO-*d*_6_ (*δ* ppm).

Position	*δ*_C_, Type	*δ*_H_ (*J* in Hz)
1, 1′	113.2, C	
2, 2′	148.9, C	
3, 3′	105.1, C	
4, 4′	144.7, C	
5, 5′	142.8, C	
6, 6′	106.6, CH	7.51, s
4, 4′-OH		8.74, br s
5, 5′-OH		9.56, br s
7, 7′	114.6, C	
8, 8′	148.7, C	
9, 9′	136.3, C	
10, 10′	130.9, C	
11, 11′	105.9, CH	6.69, s
12, 12′	149.7, C	
9, 9′-OMe	60.7, CH_3_	3.51, s
12, 12′-OMe	56.3, CH_3_	4.01, s
13, 13′	129.6, C	
14, 14′	117.2, CH	6.97, d (1.8)
15, 15′	145.1, C	
16, 16′	145.2, C	
17, 17′	115.9, CH	6.77, d (8.2)
18, 18′	120.7, CH	6.82, dd (8.2, 1.8)
15, 15′-OH		8.94, s
16, 16′-OH		8.94, s

**Table 2:** ^1^H (500 MHz) and ^13^C NMR (125 MHz) data of **2**–**4** in DMSO-*d*_6_ (*δ* ppm).

Position	2		3		4	
	*δ*_C_, Type	*δ*_H_ (*J* in Hz)	*δ*_C_, Type	*δ*_H_ (*J* in Hz)	*δ*_C_, Type	*δ*_H_ (*J* in Hz)
1	114.6, C		115.0, C		124.9, C	
2	150.4, C		149.7, C		118.5, CH	6.66, d (2.1)
3	96.8, CH	7.45, s	96.6, CH	7.38, s	145.1, C	
4	149.6, C		148.4, C		144.9, C	
5	146.6, C		144.0, C		115.6, CH	6.72, d (8.1)
6	104.4, CH	7.46, s	107.3, CH	7.39, s	121.8, CH	6.52, dd
						(8.1, 2.1)
4-OH/OMe	56.5, CH_3_	3.87, s	56.5, CH_3_	3.87, s		
5-OH/OMe	56.6, CH_3_	3.86, s		9.02, s		
1′	114.1, C		114.1, C		124.5, C	
2′	149.1, C		149.1, C		151.9, C	
3′	136.4, C		136.4, C		144.6, C	
4′	131.8, C		131.7, C		134.1, C	
5′	106.2, CH	6.73, s	106.1, CH	6.70, s	108.1, CH	6.65, s
6′	149.9, C		149.9, C		153.2, C	
2′-OMe					60.7, CH_3_	3.50, s
3′-OMe	61.1, CH_3_	3.78, s	61.0, CH_3_	3.77, s	60.7, CH_3_	3.49, s
6′-OMe	56.3, CH_3_	4.00, s	56.3, CH_3_	3.98, s	56.2, CH_3_	3.65, s
1″	129.4, C		129.5, C		128.9, C	
2″	117.2, CH	7.04, d (1.8)	117.2, CH	7.03, d (2.0)	130.4, CH	7.39, d (8.6)
3″	145.3, C		145.2, C		115.5, CH	6.83, d (8.6)
4″	145.3, C		145.3, C		157.3, C	
5″	115.9, CH	6.82, d (8.1)	115.9, CH	6.81, d (8.2)	115.5, CH	6.83, d (8.6)
6″	120.8, CH	6.89, dd	120.8, CH	6.88, dd	130.4, CH	7.39, d (8.6)
		(8.1, 1.8)		(8.2, 2.0)		
3″-OH		9.00, s				
4″-OH		9.02, s				

**Table 3:** ^1^H (500 MHz) and ^13^C NMR (125 MHz) data of **5**–**7** in DMSO-*d*_6_ (*δ* ppm).

Position	5		6		7	
	*δ*_C_, Type	*δ*_H_ (*J* in Hz)	*δ*_C_, Type	*δ*_H_ (*J* in Hz)	*δ*_C_, Type	*δ*_H_ (*J* in Hz)
1	125.3, C		134.8, C		127.2, C	
2	115.7, CH	6.85, d (1.8)	131.3, CH	7.29, d (7.1)	118.9, CH	6.73, s
3	147.2, C		127.8, CH	7.36, dd	146.3, C	
				(7.5, 7.5)		
4	145.7, C		126.7, CH	7.26, dd	145.8, C	
				(7.3, 7.3)		
5	115.2, CH	6.78, d (8.1)	127.8, CH	7.36, dd	113.5, CH	6.90, d (8.3)
				(7.5, 7.5)		
6	123.9, CH	6.71, dd	131.3, CH	7.29, d (7.1)	122.1, CH	6.64, d (8.3)
		(8.1, 1.8)				
3-OH/OMe	56.1, CH_3_	3.74, s				8.76, s
4-OH		8.87, s				
1′	118.4, C		117.1, C		117.3, C	
2′	148.7, C		148.5, C		148.6, C	
3′	139.9, C		139.7, C		139.7, C	
4′	132.9, C		133.6, C		132.9, C	
5′	103.7, CH	6.45, s	103.4, CH	6.39, s	103.4, CH	6.38, s
6′	153.6, C		153.2, C		153.5, C	
2′-OH		8.58, s		8.61, s		8.49, s
3′-OMe	60.8, CH_3_	3.30, s	60.5, CH_3_	3.32, s	60.5, CH_3_	3.30, s
6′-OMe	56.1, CH_3_	3.66, s	56.0, CH_3_	3.64, s	56.0, CH_3_	3.64, s
1″	138.7, C		129.6, C		129.1, C	
2″	129.1, CH	7.60, d (7.2)	116.5, CH	7.06, d (1.9)	130.1, CH	7.43, d (8.5)
3″	128.8, CH	7.46, dd	145.4, C		115.6, CH	6.84, d (8.5)
		(7.5, 7.5)				
4″	127.6, CH	7.37, dd	145.3, C		157.2, C	
		(7.4, 7.4)				
5″	128.8, CH	7.46, dd	116.0, CH	6.81, d (8.1)	115.6, CH	6.84, d (8.5)
		(7.5, 7.5)				
6″	129.1, CH	7.60, d (7.2)	120.1, C	6.90, dd	130.1, CH	7.43, d (8.5)
				(8.1, 1.9)		
3″-OH				8.98, s		
4″-OH				9.00, s		9.51, s
1‴					65.3, CH_2_	4.62, d (6.2)
2‴					119.3, CH	5.71, t (6.2)
3‴					140.2, C	
4‴					14.3, CH_3_	1.67, s
5‴					66.0, CH_3_	3.86, d (4.4)
5‴-OH						4.89, t (4.4)

## Data Availability

Data is contained within the article or supplementary material. The data presented in this study are available in article or supplementary material.

## Supplementary Materials

The following are available online at https://www.mdpi.com/1660-3397/19/2/82/s1, Figures S1–S51: 1D and 2D NMR and HRESIMS spectra of **1**–**7**, Figure S52: HPLC of LDJ-5 crude extract, Figure S53: Chiral HPLC analysis of **1**, Figures S54–S60: IR spectra of **1**–**7**, Table S1: Antimicrobial activity of **1**–**7**.
