# Supplementary material for: Polyhydroxy p-Terphenyls from a Mangrove Endophytic Fungus Aspergillus candidus LDJ-5
Source: Mar Drugs. 2021 Feb 2;19(2):82. doi: 10.3390/md19020082 (PMC7912881; doi:10.3390/md19020082)
Supplement: Supplementary file 1 [file marinedrugs-19-00082-s001.pdf]

Supporting Information

Polyhydroxy *p*-Terphenyls from a Mangrove Endophytic Fungus *Aspergillus candidus* LDJ-5

Guoliang Zhou,[1] Xiaomin Zhang,[1] Mudassir Shah, [1] Qian Che,[1] Guojian Zhang,[1,2] Qianqun Gu,[1] Tianjiao Zhu,[*,1] and Dehai Li[*,1,2,3]

[1] Key Laboratory of Marine Drugs, Chinese Ministry of Education, School of Medicine and Pharmacy, Ocean University of China, Qingdao 266003, People's Republic of China

[2] Laboratory for Marine Drugs and Bioproducts, Pilot National Laboratory for Marine Science and Technology, Qingdao, 266237, P. R. China

[3] Open Studio for Druggability Research of Marine Natural Products, Pilot National Laboratory for Marine Science and Technology, Qingdao, 266237, People's Republic of China

[*] Corresponding authors: Tel: 0086-532-82031619. E-mail: dehaili@ouc.edu.cn (D. Li); zhutj@ouc.edu.cn (T. Zhu).

List of supporting information

Figure S1. ^{1}H NMR (500 MHz, DMSO-d_6) spectrum of **1**........S5

Figure S2. ^{13}C NMR (125 MHz, DMSO-d_6) spectrum of **1**. S5

Figure S3. DEPT (125 MHz, DMSO-d_6) spectrum of **1**. S6

Figure S4. HSQC spectrum of **1** in DMSO-d_6........S6

Figure S5. ^{1}H-^{1}H COSY spectrum of **1** in DMSO-d_6. ....S7

Figure S6. HMBC spectrum of **1** in DMSO-d_6........S7

Figure S7. NOESY spectrum of **1** in DMSO-d_6. ....S8

Figure S8. HRESIMS spectrum of **1**........S8

Figure S9. ^{1}H NMR (500 MHz, DMSO-d_6) spectrum of **2**........S9

Figure S10. ^{13}C NMR (125 MHz, DMSO-d_6) spectrum of **2**........S9

Figure S11. DEPT (125 MHz, DMSO-d_6) spectrum of **2**. S10

Figure S12. HSQC spectrum of **2** in DMSO-d_6........S10

Figure S13. ^{1}H-^{1}H COSY spectrum of **2** in DMSO-d_6. ....S11

Figure S14. HMBC spectrum of **2** in DMSO-d_6........S11

Figure S15. HRESIMS spectrum of **2**........S12

Figure S16. ^{1}H NMR (500 MHz, DMSO-d_6) spectrum of **3**........S12

Figure S17. ^{13}C NMR (125 MHz, DMSO-d_6) spectrum of **3**........S13

Figure S18. DEPT (125 MHz, DMSO-d_6) spectrum of **3**.. ....S13

Figure S19. HSQC spectrum of **3** in DMSO-d_6........S14

Figure S20. ^{1}H-^{1}H COSY spectrum of **3** in DMSO-d_6. ....S14

Figure S21. HMBC spectrum of **3** in DMSO-d_6........S15

Figure S22. HRESIMS spectrum of **3**........S15

Figure S23. ^{1}H NMR (500 MHz, DMSO-d_6) spectrum of **4**........S16

Figure S24. ^{13}C NMR (125 MHz, DMSO-d_6) spectrum of **4**........S16

Figure S25. DEPT (125 MHz, DMSO-d_6) spectrum of **4**.. ....S17

Figure S26. HSQC spectrum of **4** in DMSO-d_6........S17

Figure S27. ^{1}H-^{1}H COSY spectrum of **4** in DMSO-d_6. ....S18

Figure S28. HMBC spectrum of **4** in DMSO-d_6. ....S18

Figure S29. HRESIMS spectrum of **4** S19

Figure S30. ^{1}H NMR (500 MHz, DMSO-d_6) spectrum of **5**....S19

Figure S31. ^{13}C NMR (125 MHz, DMSO-d_6) spectrum of **5**....S20

Figure S32. DEPT (125 MHz, DMSO-d_6) spectrum of **5**.. ....S20

Figure S33. HSQC spectrum of **5** in DMSO-d_6. ....S21

Figure S34. ^{1}H-^{1}H COSY spectrum of **5** in DMSO-d_6. ....S21

Figure S35. HMBC spectrum of **5** in DMSO-d_6. ....S22

Figure S36. HRESIMS spectrum of **5** S22

Figure S37. ^{1}H NMR (500 MHz, DMSO-d_6) spectrum of **6**....S23

Figure S38. ^{13}C NMR (125 MHz, DMSO-d_6) spectrum of **6**....S23

Figure S39. DEPT (125 MHz, DMSO-d_6) spectrum of **6**.. ....S24

Figure S40. HSQC spectrum of **6** in DMSO-d_6. ....S24

Figure S41. ^{1}H-^{1}H COSY spectrum of **6** in DMSO-d_6. ....S25

Figure S42. HMBC spectrum of **6** in DMSO-d_6. ....S25

Figure S43. HRESIMS spectrum of **6** S26

Figure S44. ^{1}H NMR (500 MHz, DMSO-d_6) spectrum of **7**....S26

Figure S45. ^{13}C NMR (125 MHz, DMSO-d_6) spectrum of **7**....S27

Figure S46. DEPT (125 MHz, DMSO-d_6) spectrum of **7**.. ....S27

Figure S47. HSQC spectrum of **7** in DMSO-d_6. ....S28

Figure S48. ^{1}H-^{1}H COSY spectrum of **7** in DMSO-d_6. ....S28

Figure S49. HMBC spectrum of **7** in DMSO-d_6. ....S29

Figure S50. NOESY spectrum of **7** in DMSO-d_6. ....S29

Figure S51. HRESIMS spectrum of **7** S30

Figure S52. HPLC of LDJ-5 crude extract. S30

Figure S53. Chiral HPLC analysis of **1**.. ....S31

Figure S54. IR spectrum of **1** S31

Figure S55. IR spectrum of **2** S31

Figure S56. IR spectrum of **3**. S32

Figure S57. IR spectrum of **4** S32

Figure S58. IR spectrum of **5**. S32

Figure S59. IR spectrum of **6**. S33

Figure S60. IR spectrum of **7**. S33

Table S1. Antimicrobial activity of **1-7** S33

Figure S1. ^{1}H NMR (500 MHz, DMSO-d_6) spectrum of **1**.

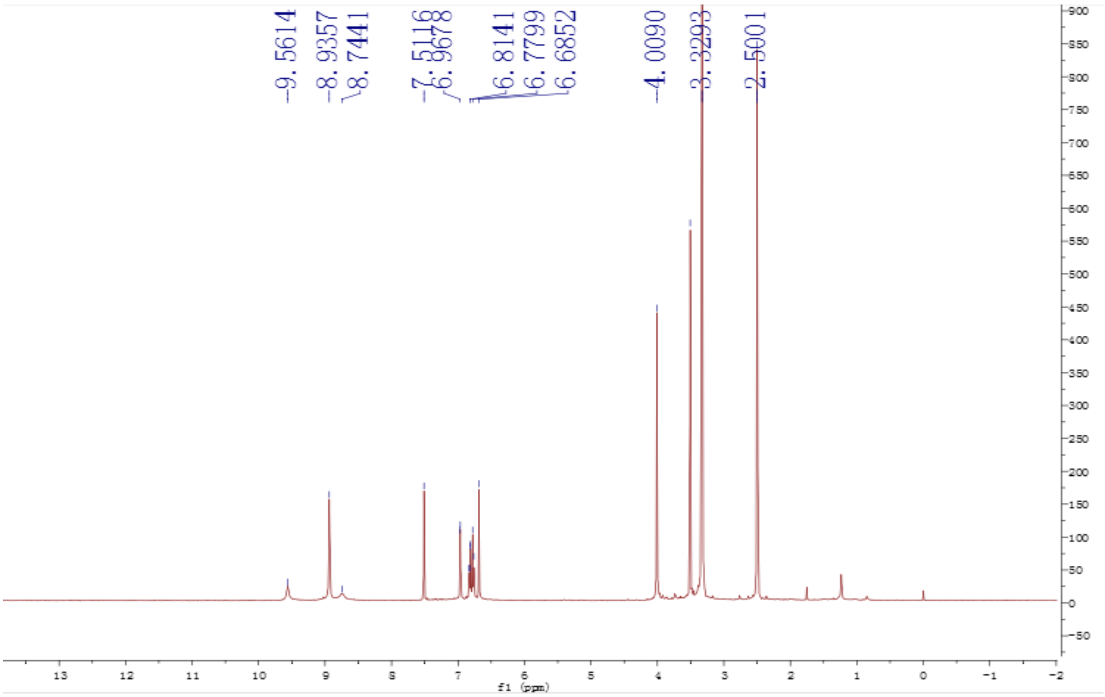

Figure S2. ^{13}C NMR (125 MHz, DMSO-d_6) spectrum of **1**.

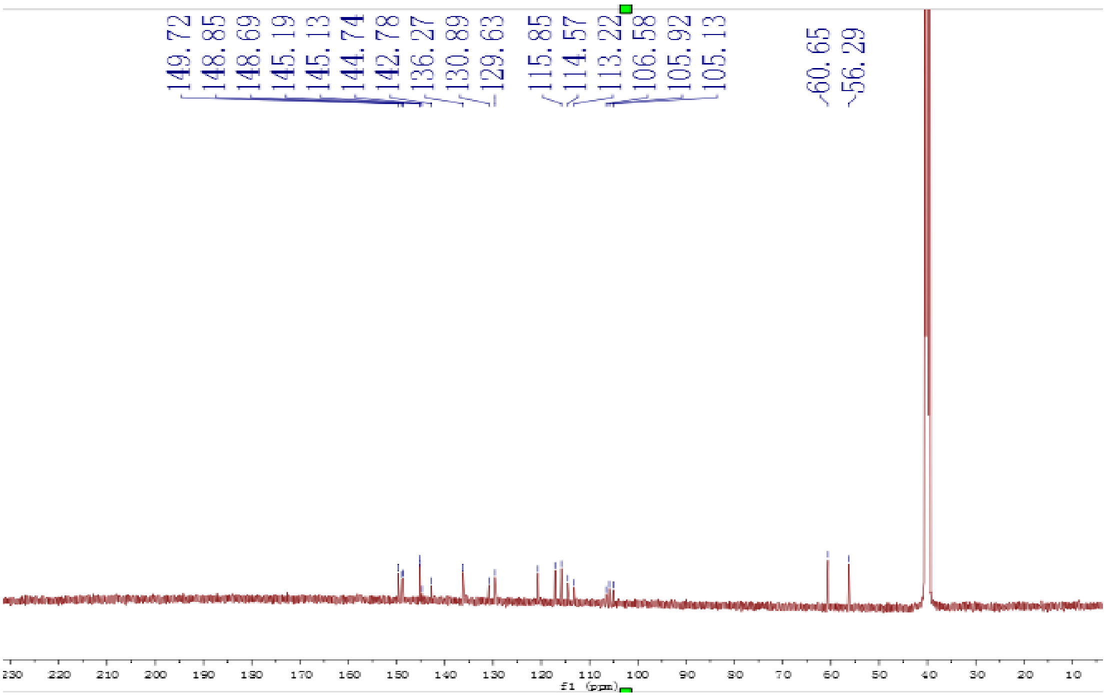

Figure S3. DEPT (125 MHz, DMSO-d_6) spectrum of **1**.

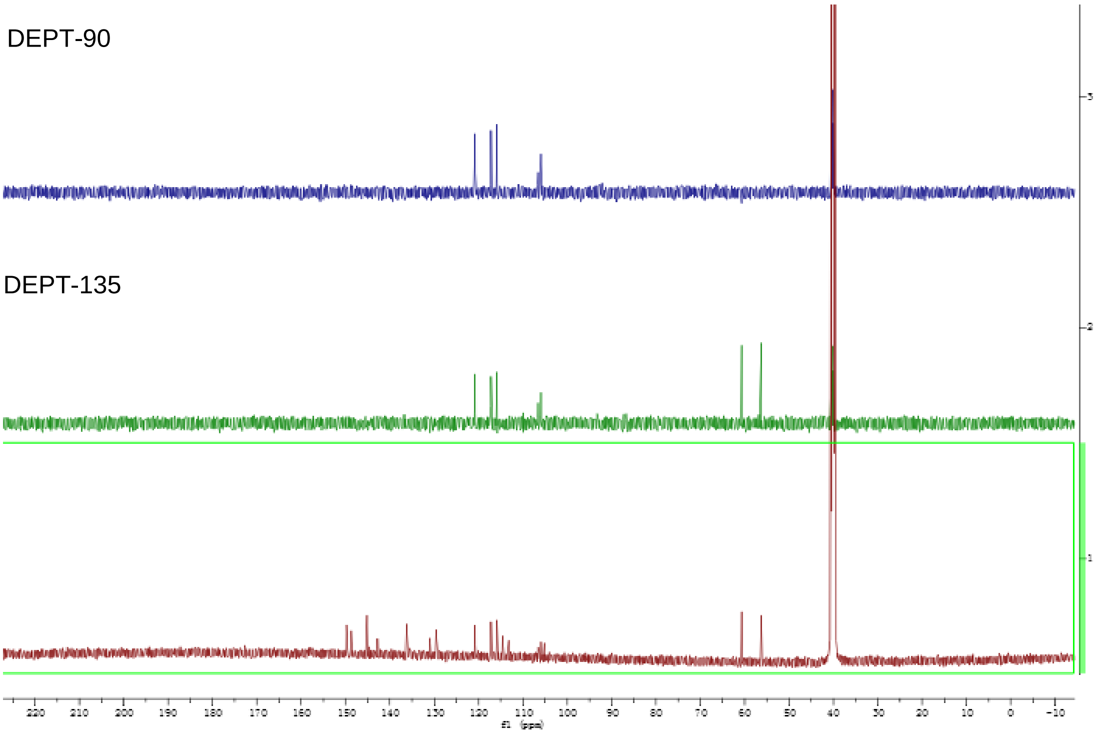

Figure S4. HSQC spectrum of **1** in DMSO-d_6.

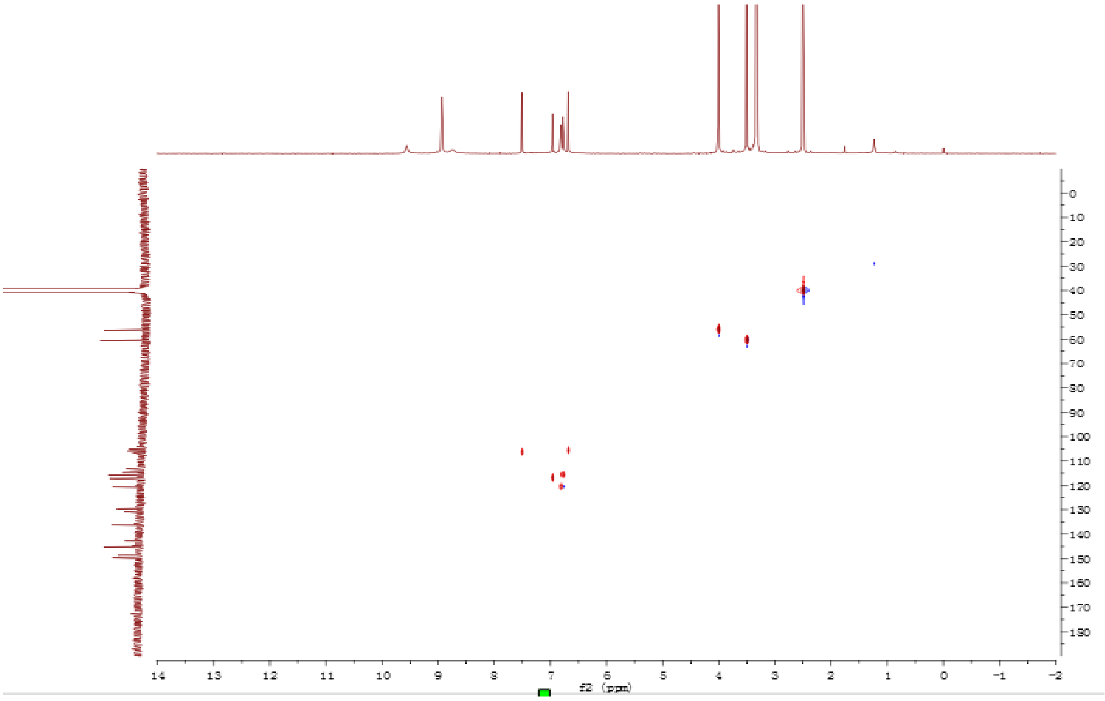

Figure S5. ^{1}H-^{1}H COSY spectrum of **1** in DMSO-d_6.

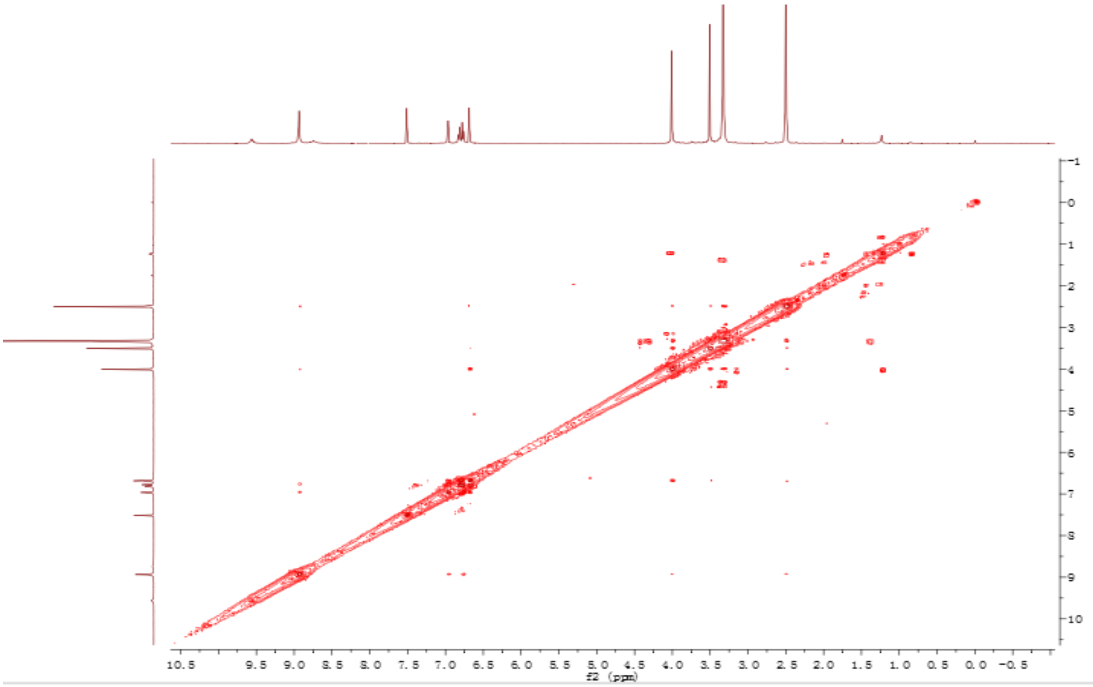

Figure S6. HMBC spectrum of **1** in DMSO-d_6.

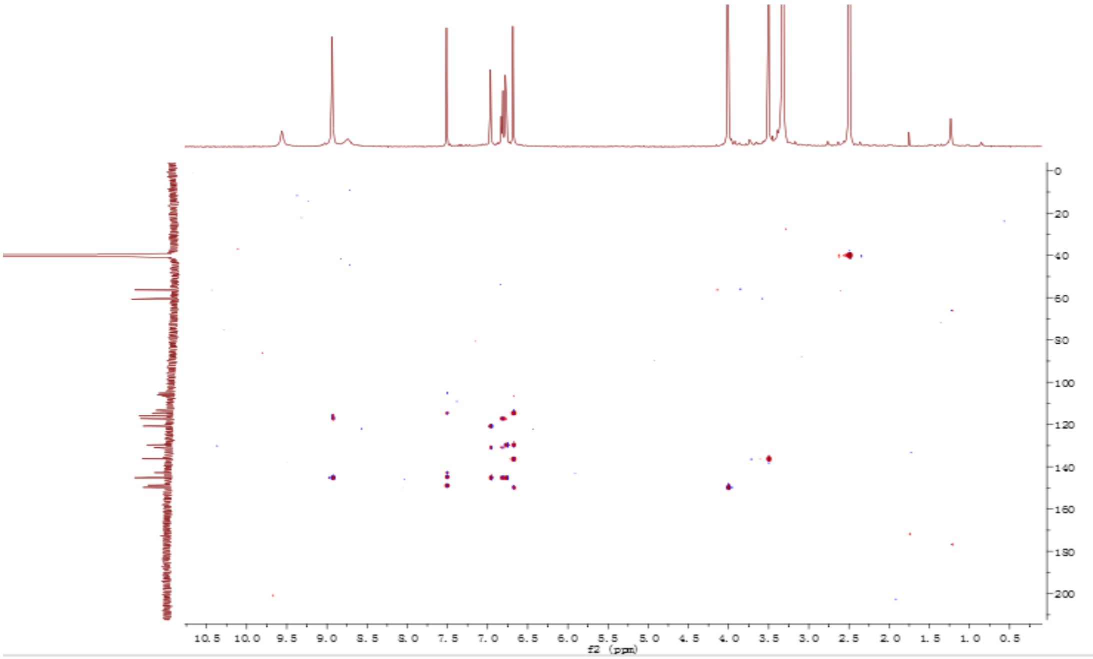

Figure S7. NOESY spectrum of **1** in DMSO-d_6.

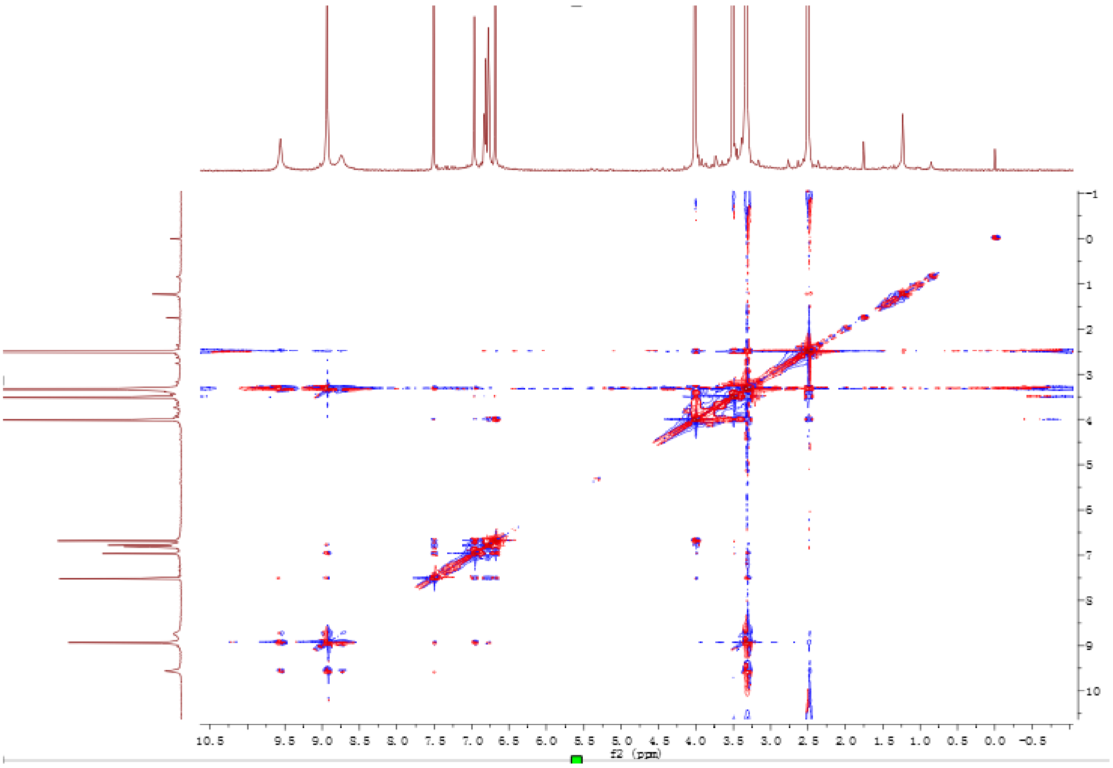

Figure S8. HRESIMS spectrum of **1**.

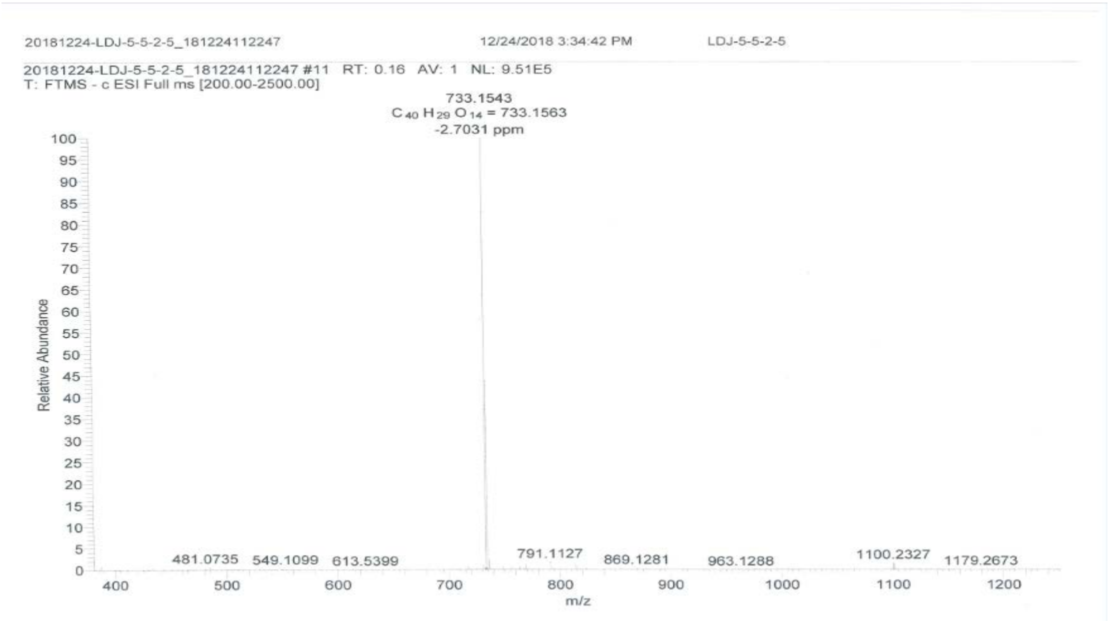

Figure S9. ^{1}H NMR (500 MHz, DMSO-d_6) spectrum of **2**.

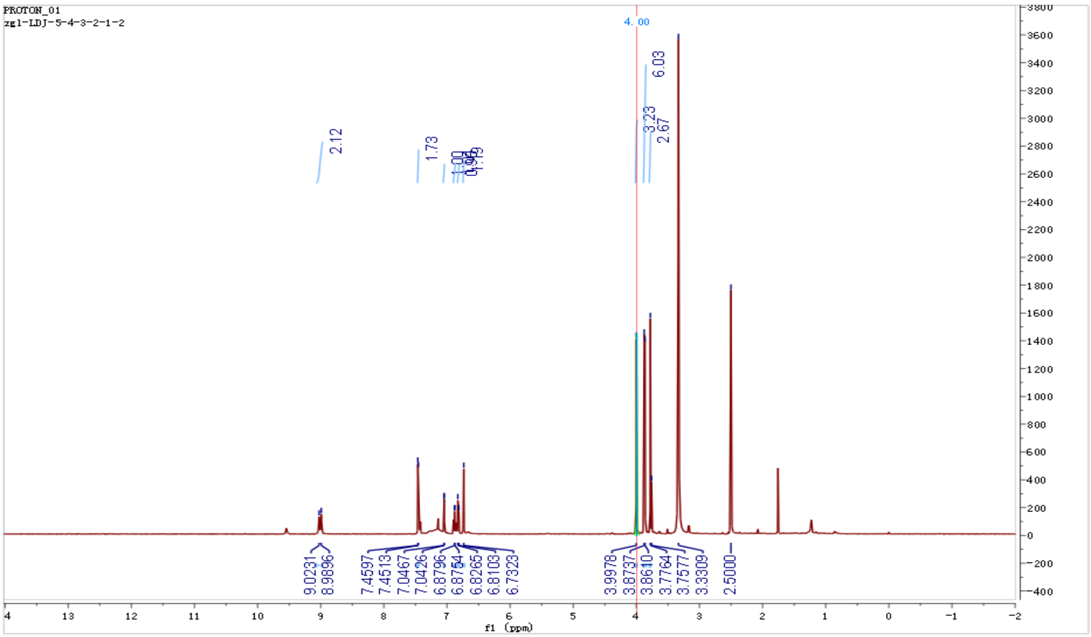

Figure S10. ^{13}C NMR (125 MHz, DMSO-d_6) spectrum of **2**.

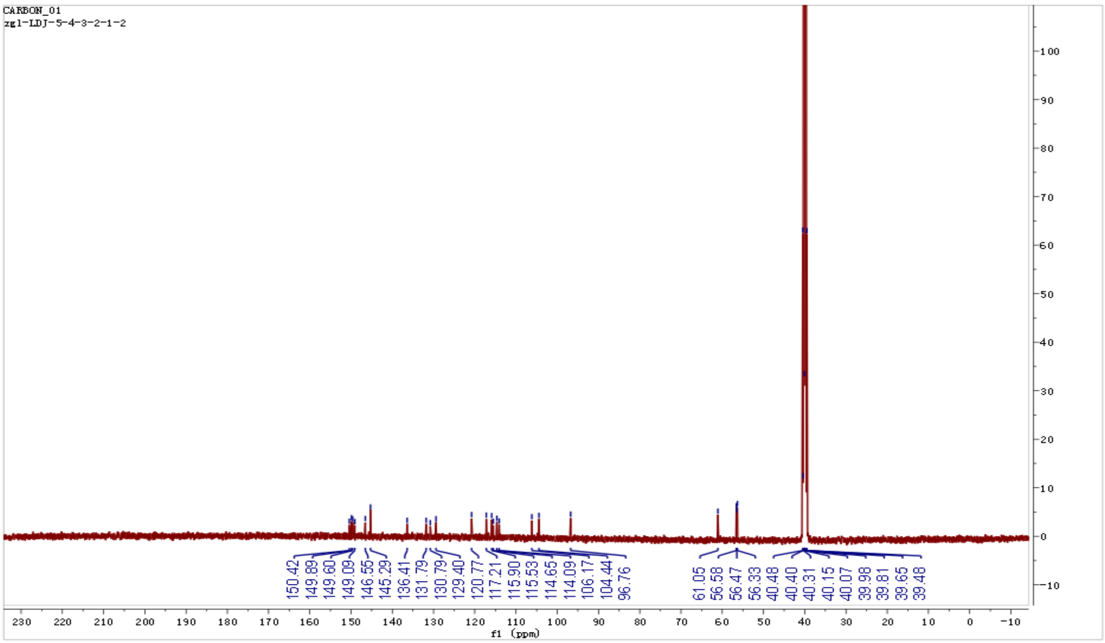

Figure S11. DEPT (125 MHz, DMSO-d_6) spectrum of **2**.

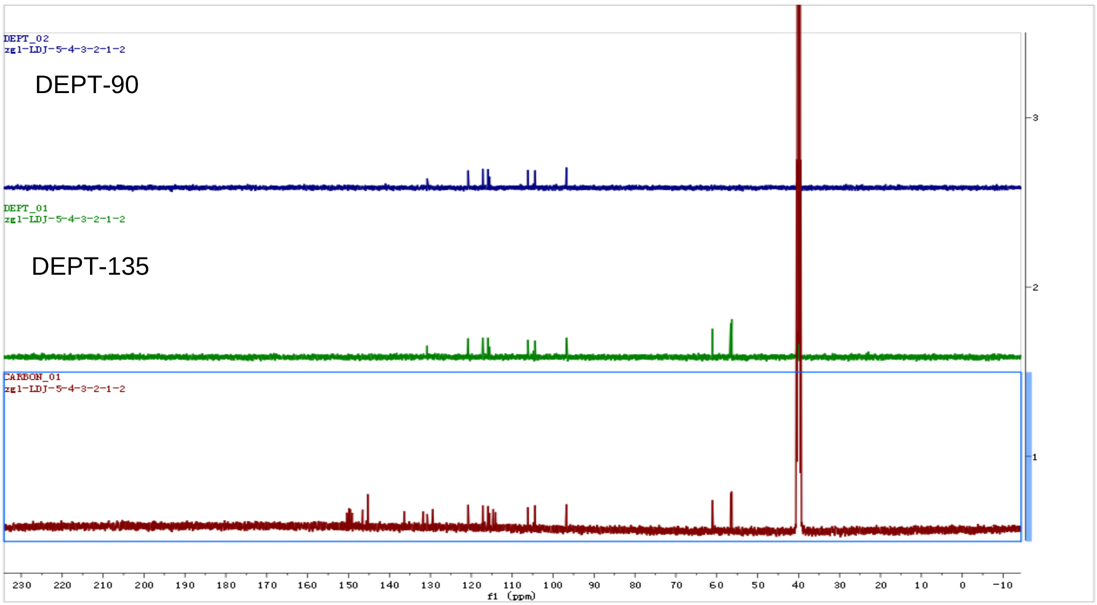

Figure S12. HSQC spectrum of **2** in DMSO-d_6.

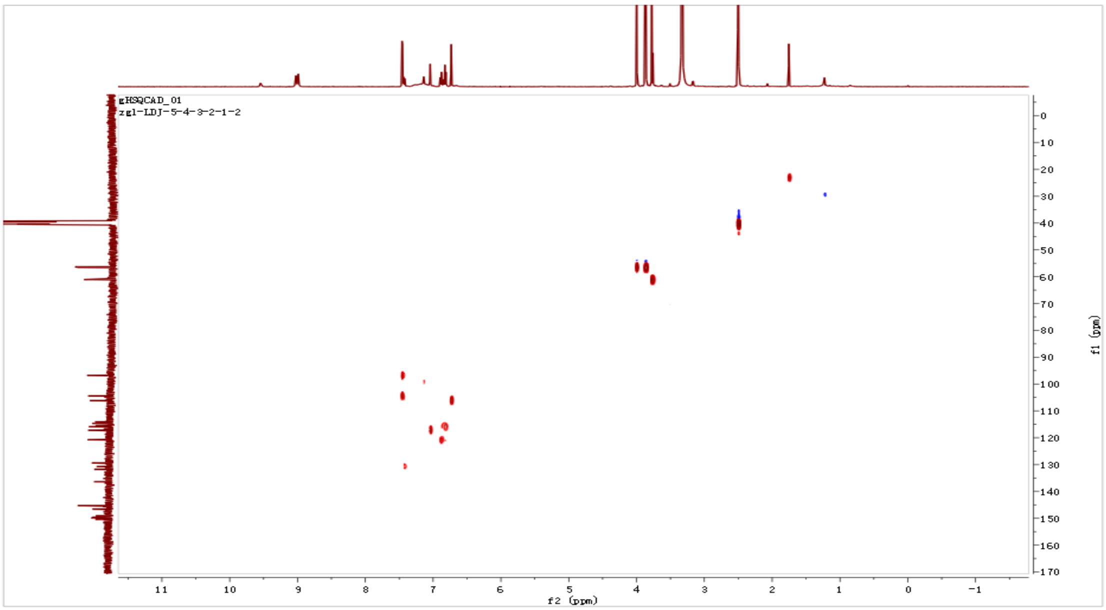

Figure S13. ^{1}H-^{1}H COSY spectrum of **2** in DMSO-d_6.

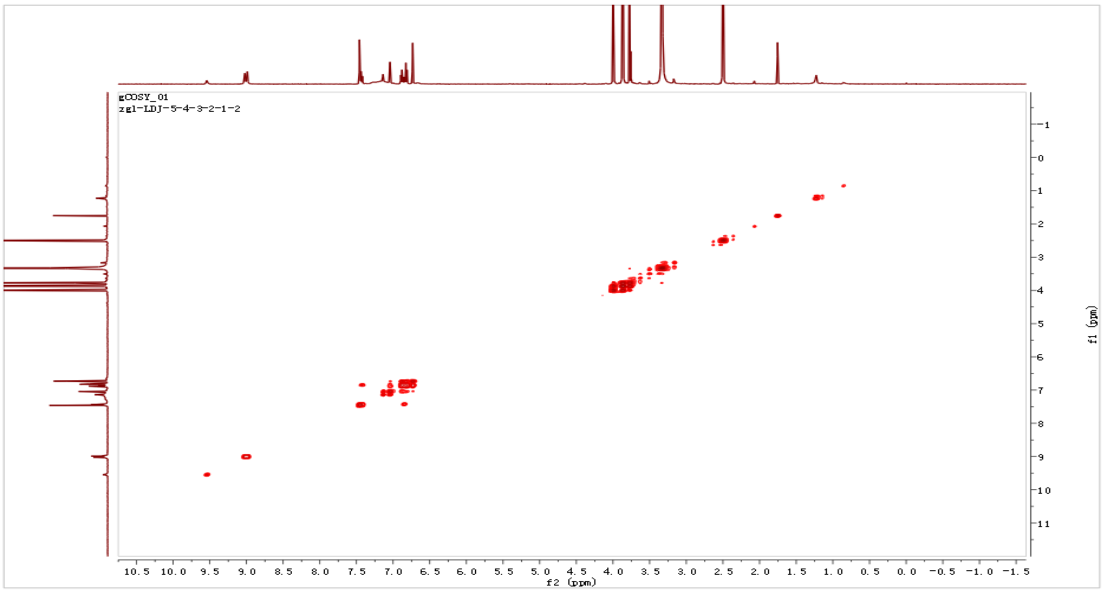

Figure S14. HMBC spectrum of **2** in DMSO-d_6.

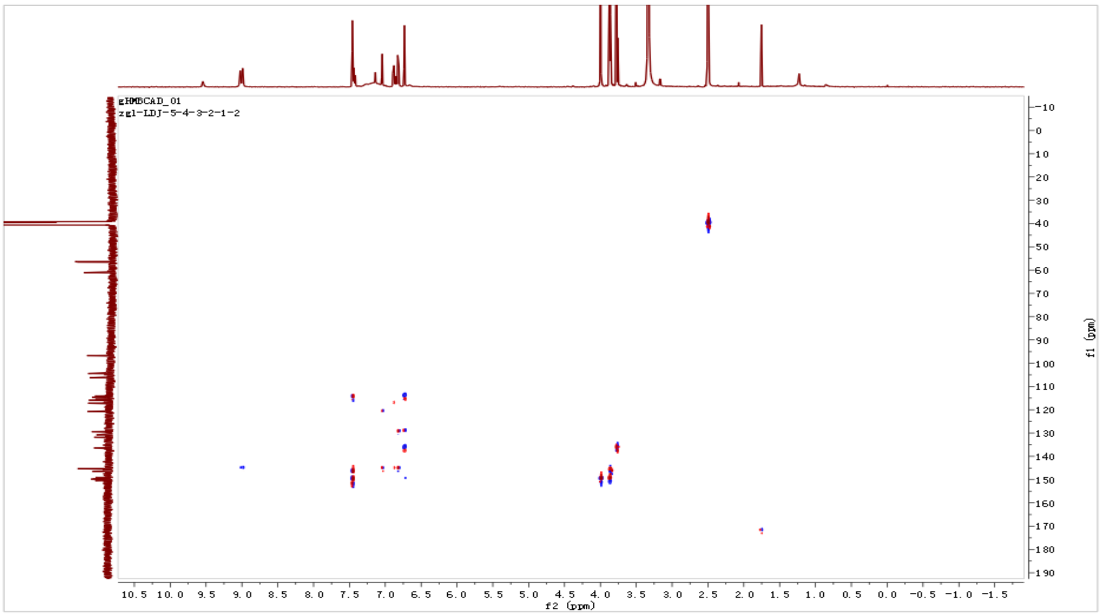

Figure S15. HRESIMS spectrum of **2**.

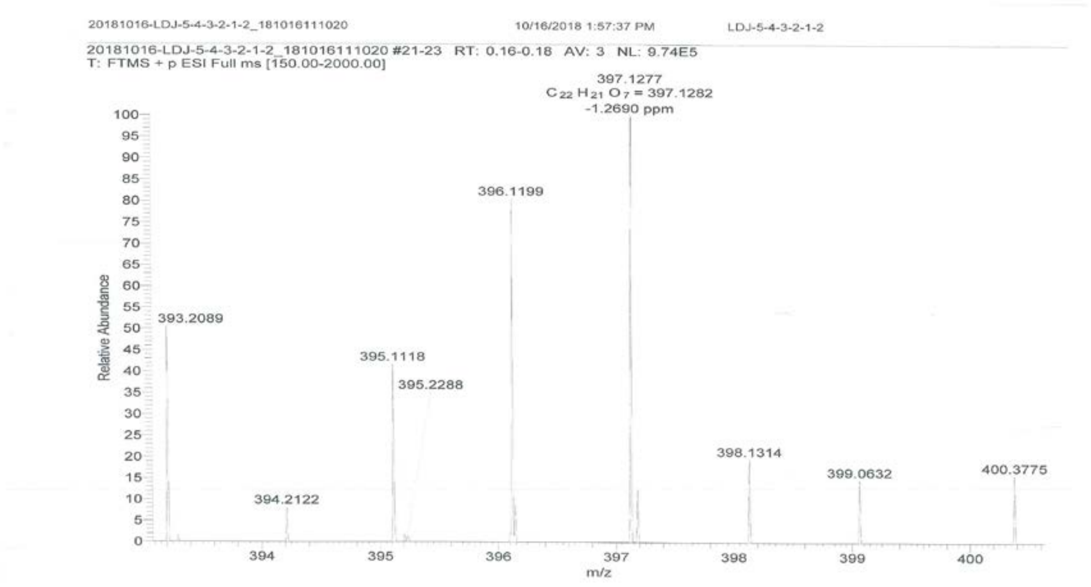

Figure S16. ^{1}H NMR (500 MHz, DMSO-d_6) spectrum of **3**.

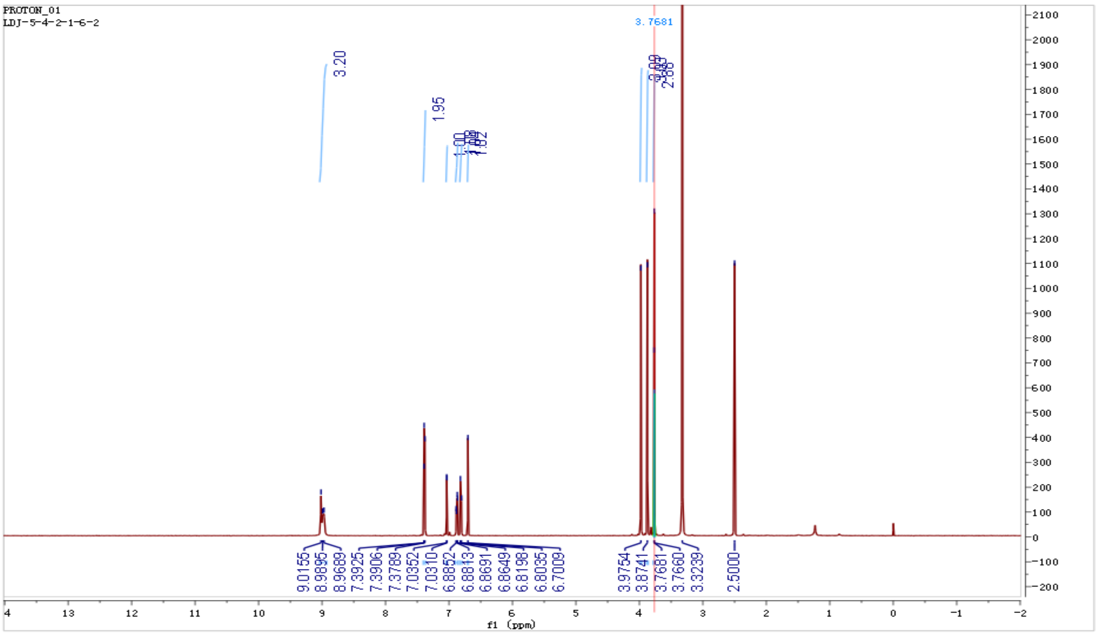

Figure S17. ^{13}C NMR (125 MHz, DMSO-d_6) spectrum of **3**.

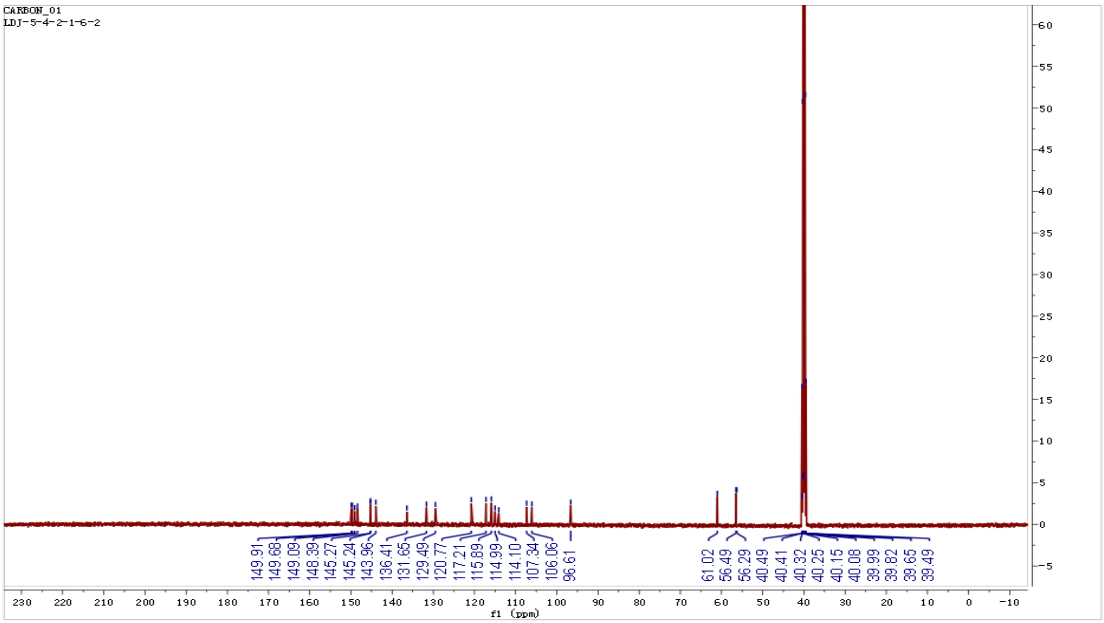

Figure S18. DEPT (125 MHz, DMSO-d_6) spectrum of **3**.

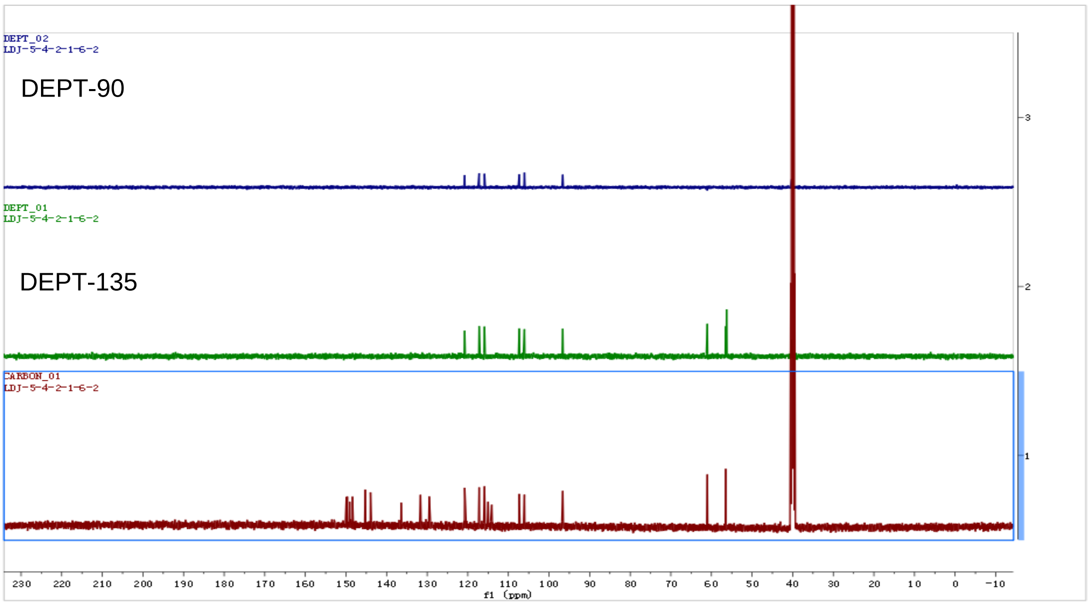

Figure S19. HSQC spectrum of **3** in DMSO-d_6.

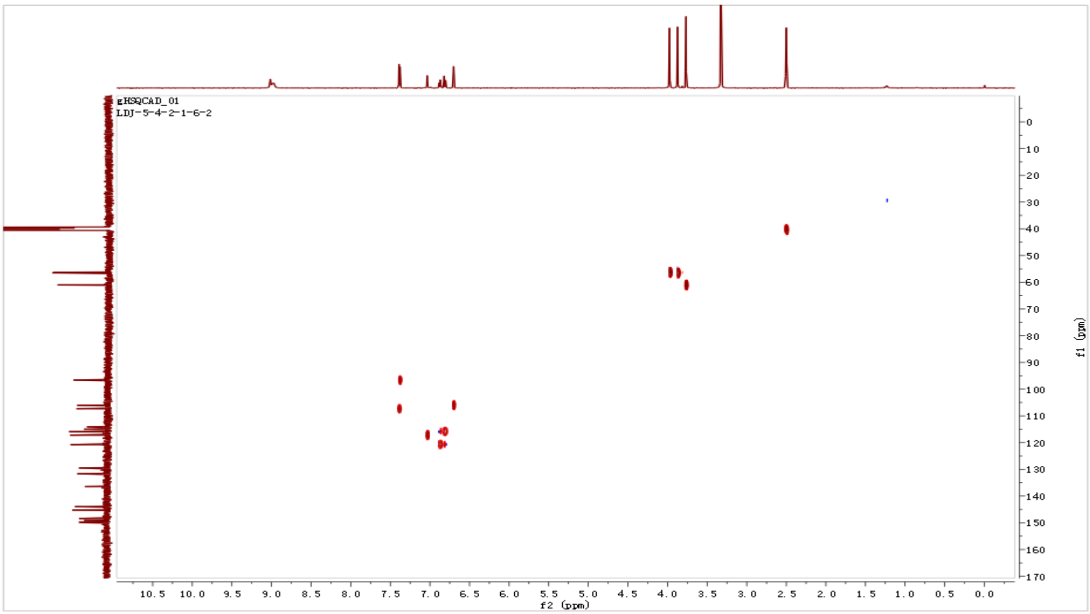

Figure S20. ^{1}H-^{1}H COSY spectrum of **3** in DMSO-d_6.

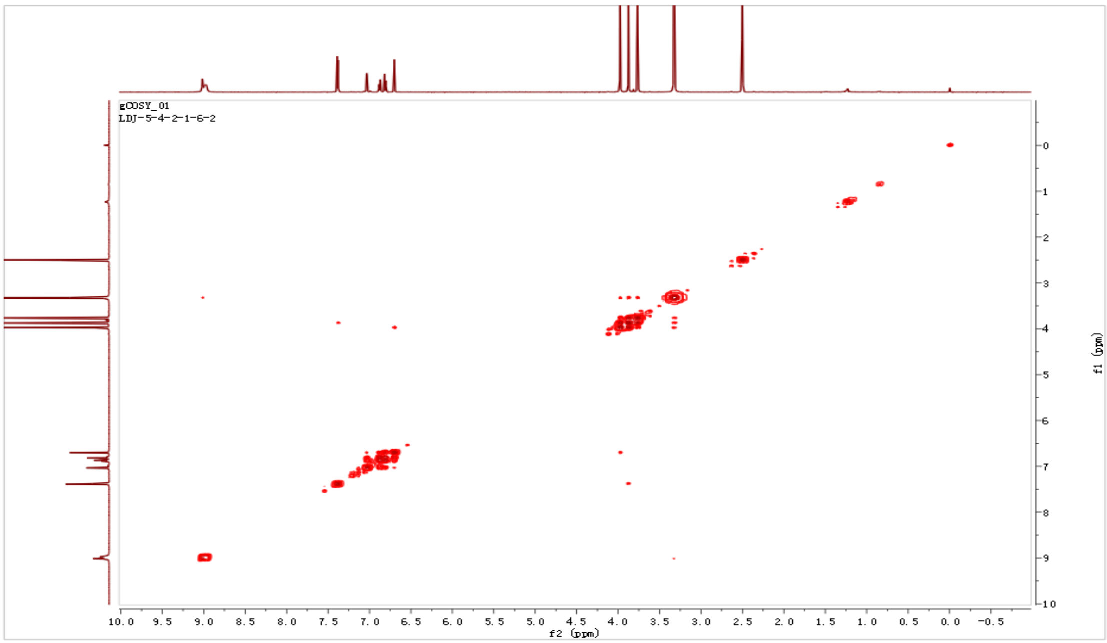

Figure S21. HMBC spectrum of **3** in DMSO-d_6.

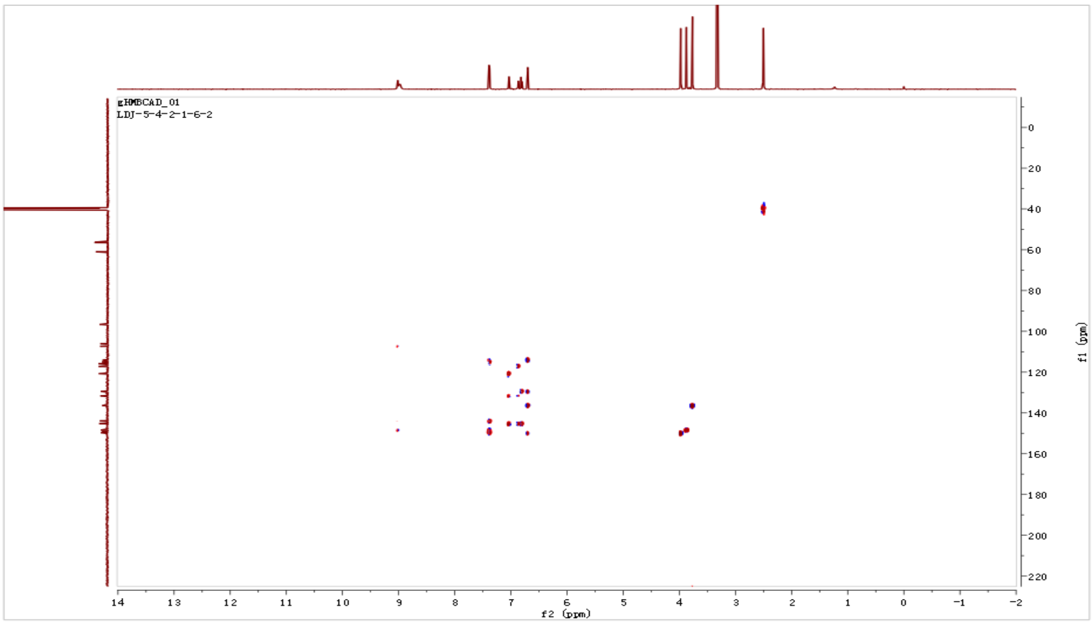

Figure S22. HRESIMS spectrum of **3**.

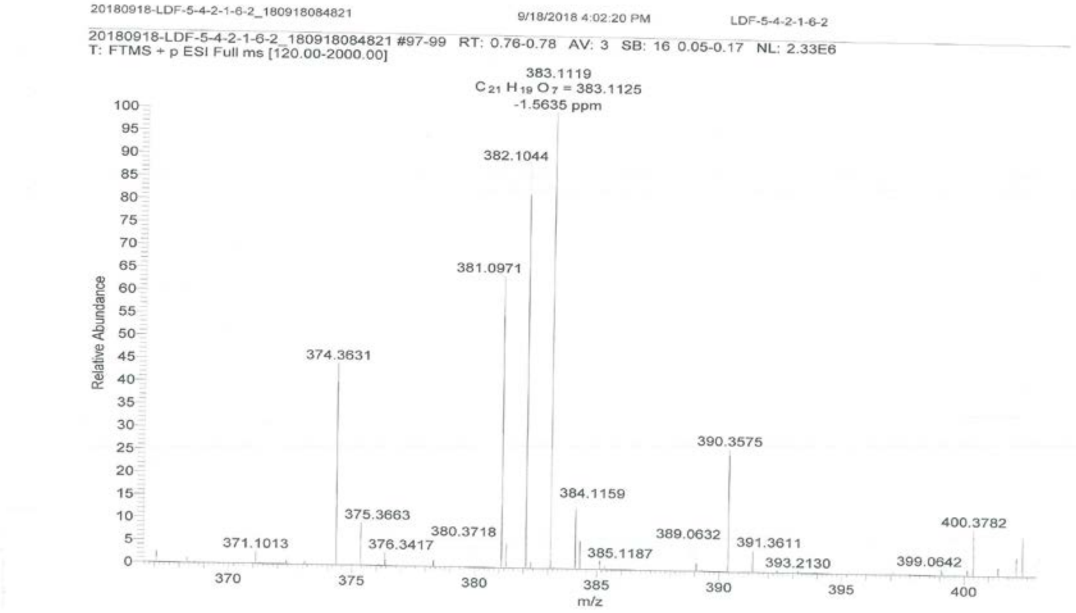

Figure S23. ^{1}H NMR (500 MHz, DMSO-d_6) spectrum of **4**.

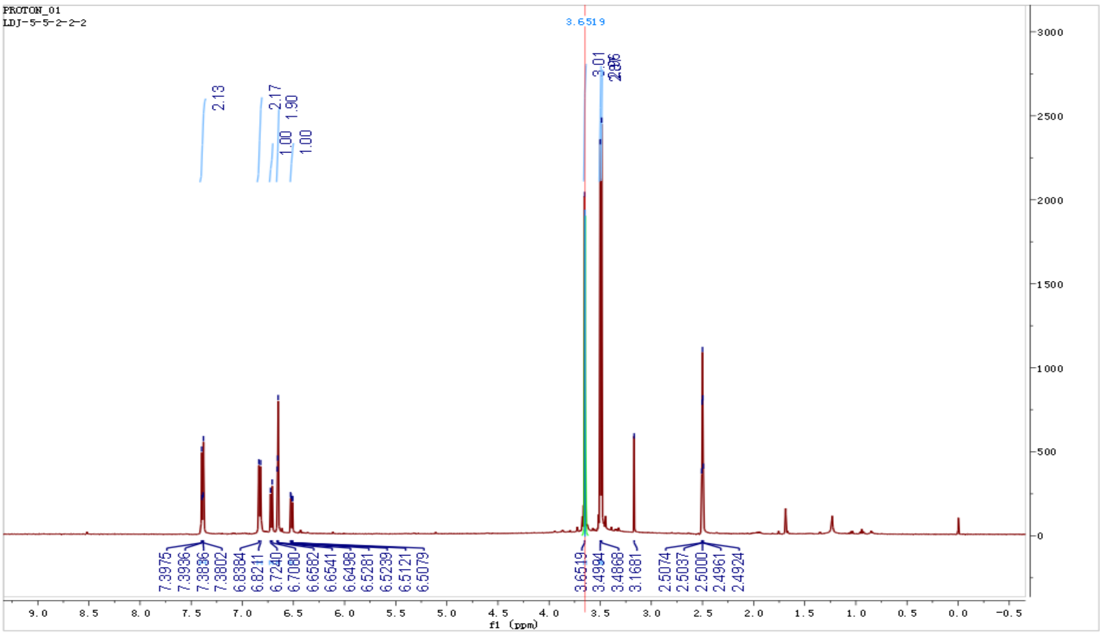

Figure S24. ^{13}C NMR (125 MHz, DMSO-d_6) spectrum of **4**.

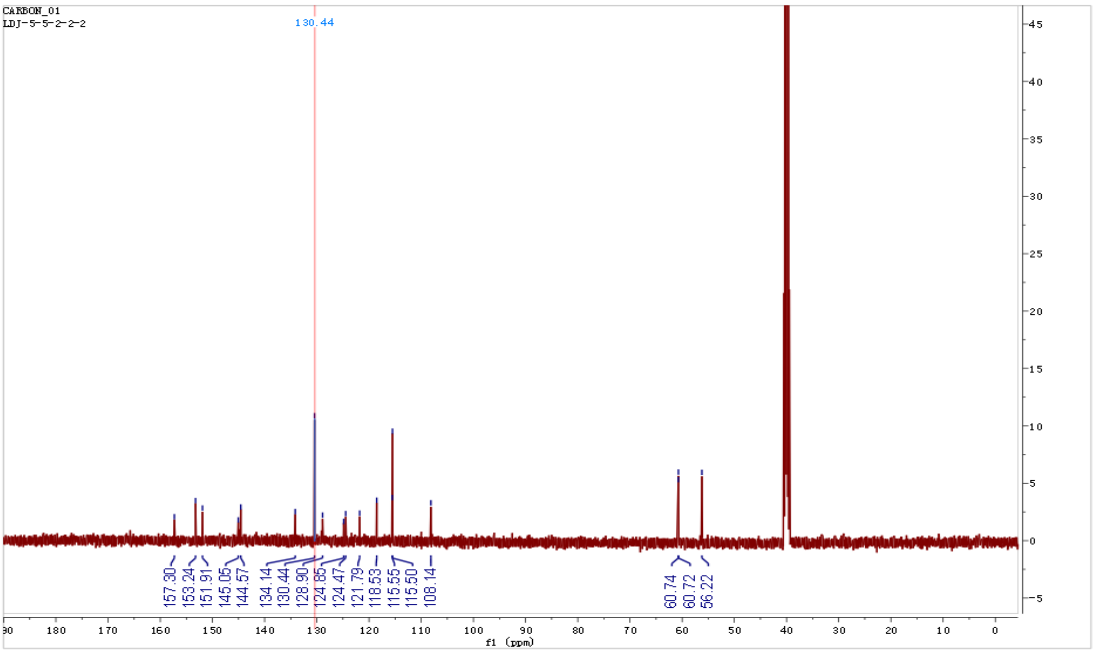

Figure S25. DEPT (125 MHz, DMSO-d_6) spectrum of **4**.

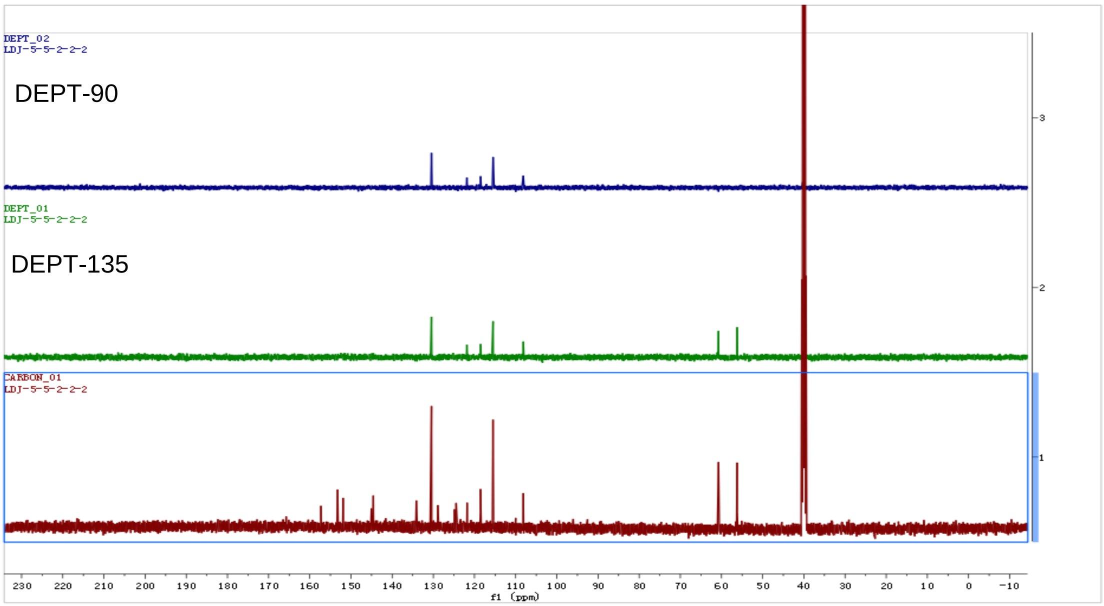

Figure S26. HSQC spectrum of **4** in DMSO-d_6.

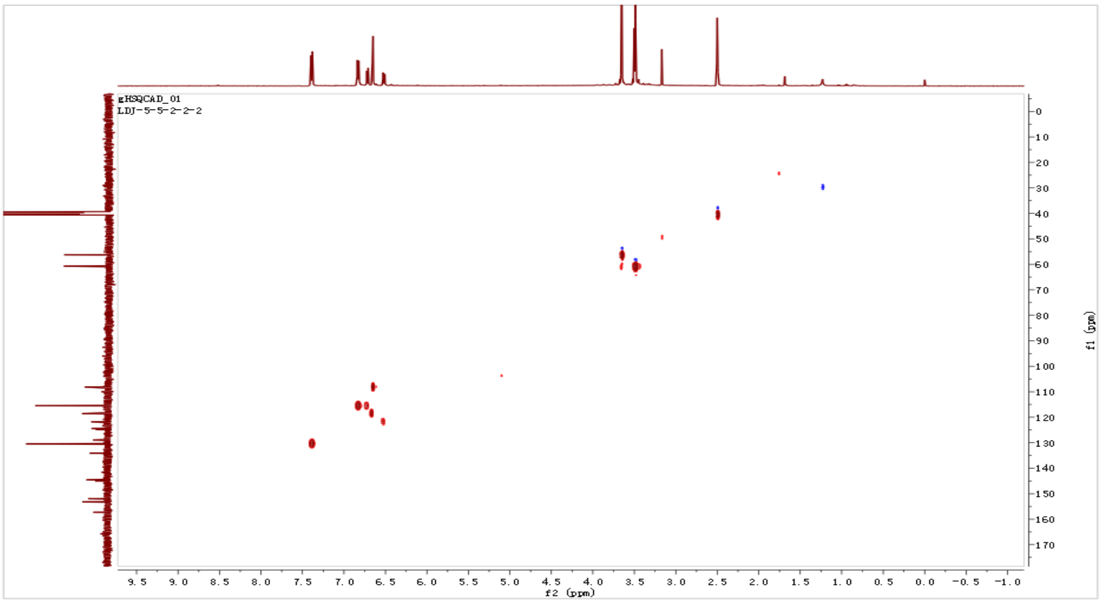

Figure S27. ^{1}H-^{1}H COSY spectrum of **4** in DMSO-d_6.

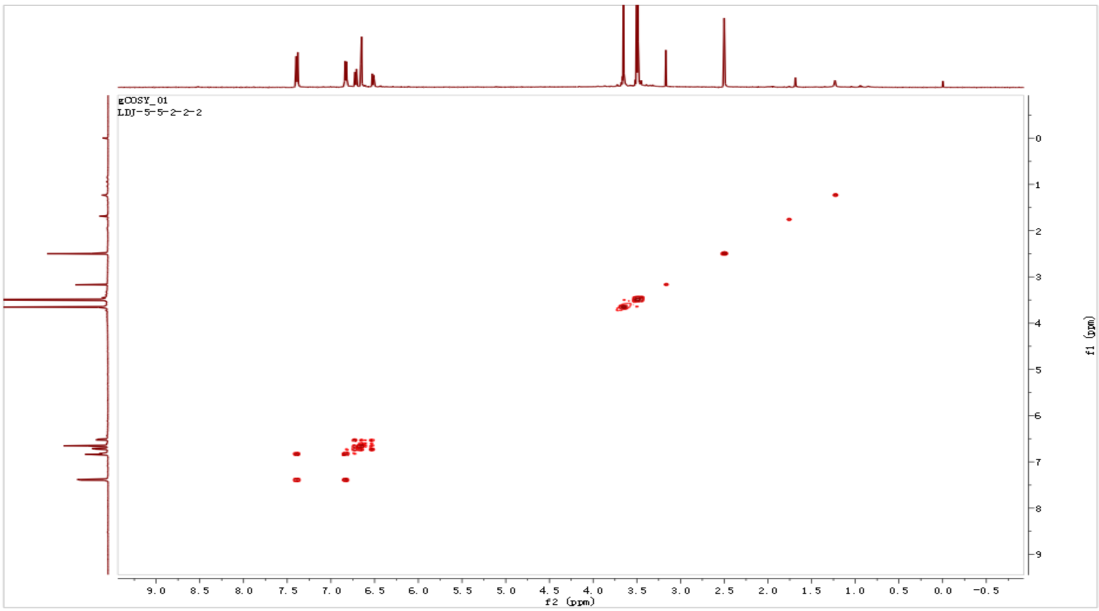

Figure S28. HMBC spectrum of **4** in DMSO-d_6.

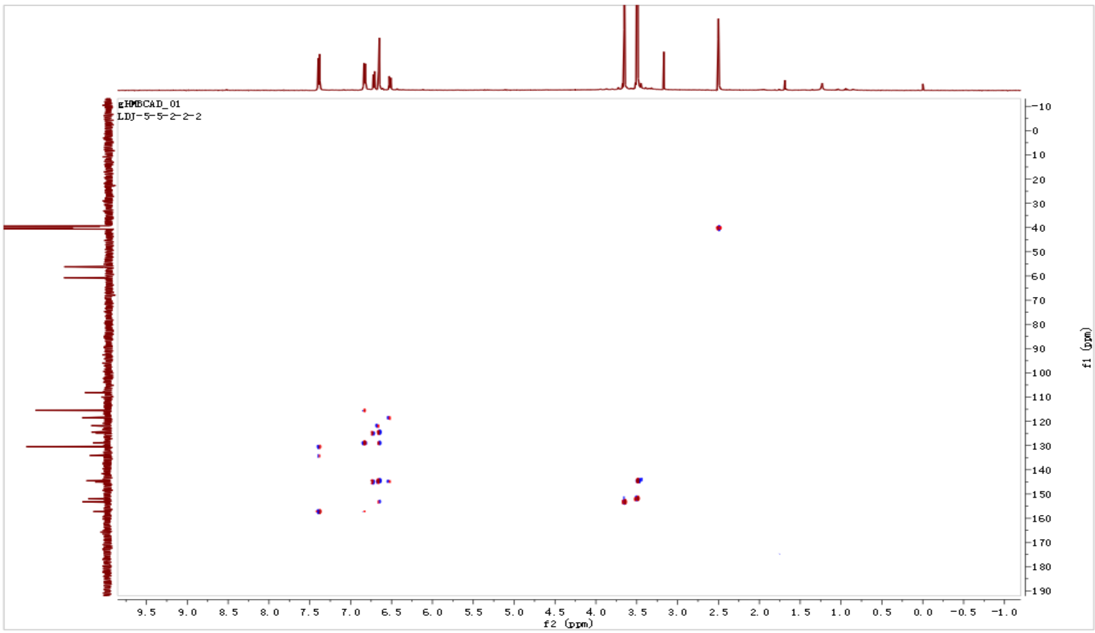

Figure S29. HRESIMS spectrum of **4**.

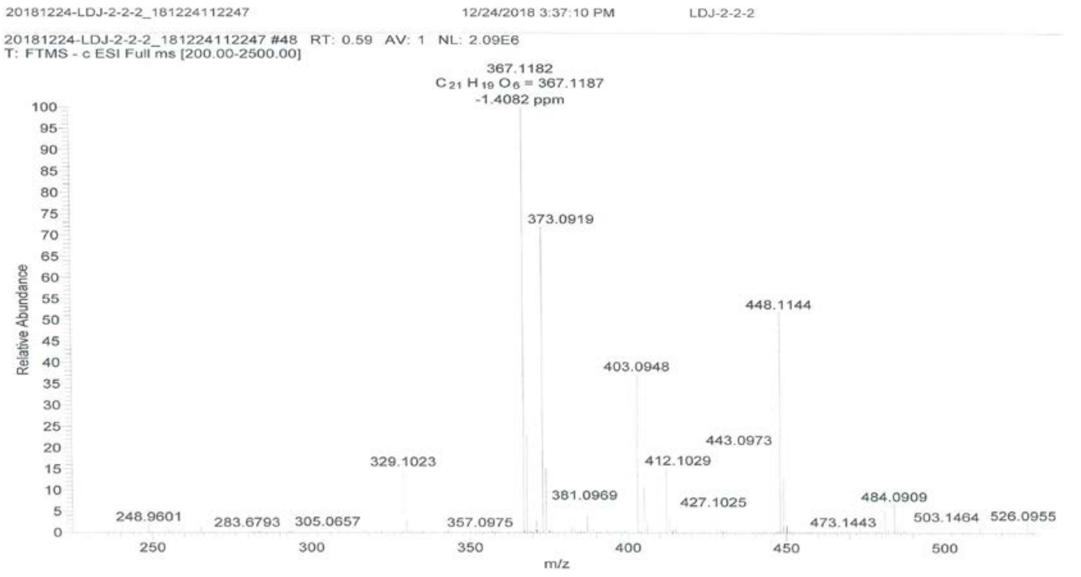

Figure S30. ^{1}H NMR (500 MHz, DMSO-d_6) spectrum of **5**.

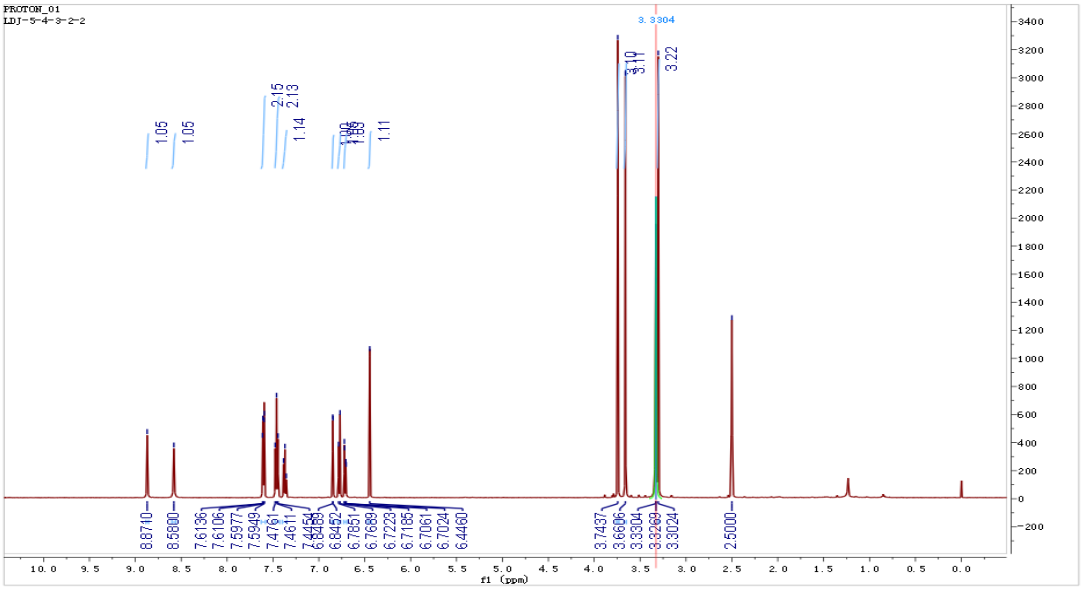

Figure S31. ^{13}C NMR (125 MHz, DMSO-d_6) spectrum of **5**.

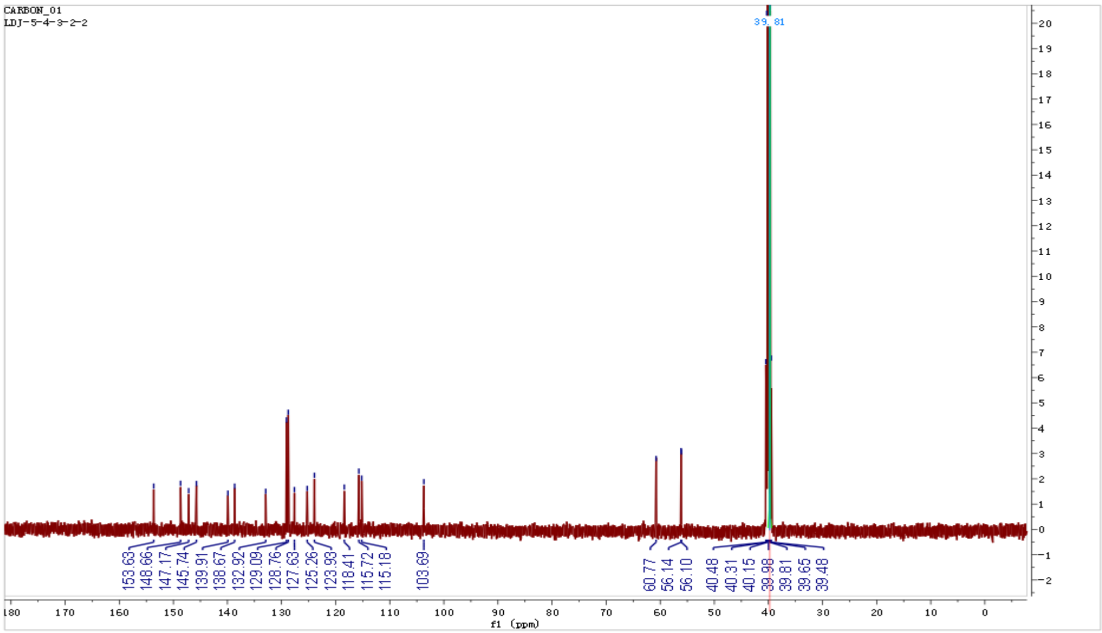

Figure S32. DEPT (125 MHz, DMSO-d_6) spectrum of **5**.

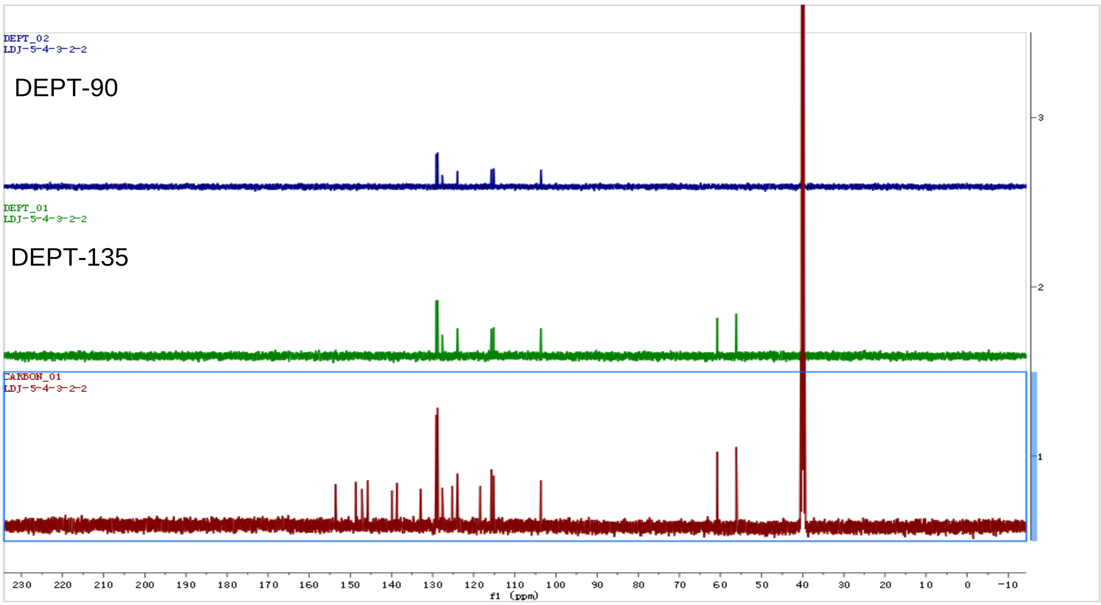

Figure S33. HSQC spectrum of **5** in DMSO-d_6.

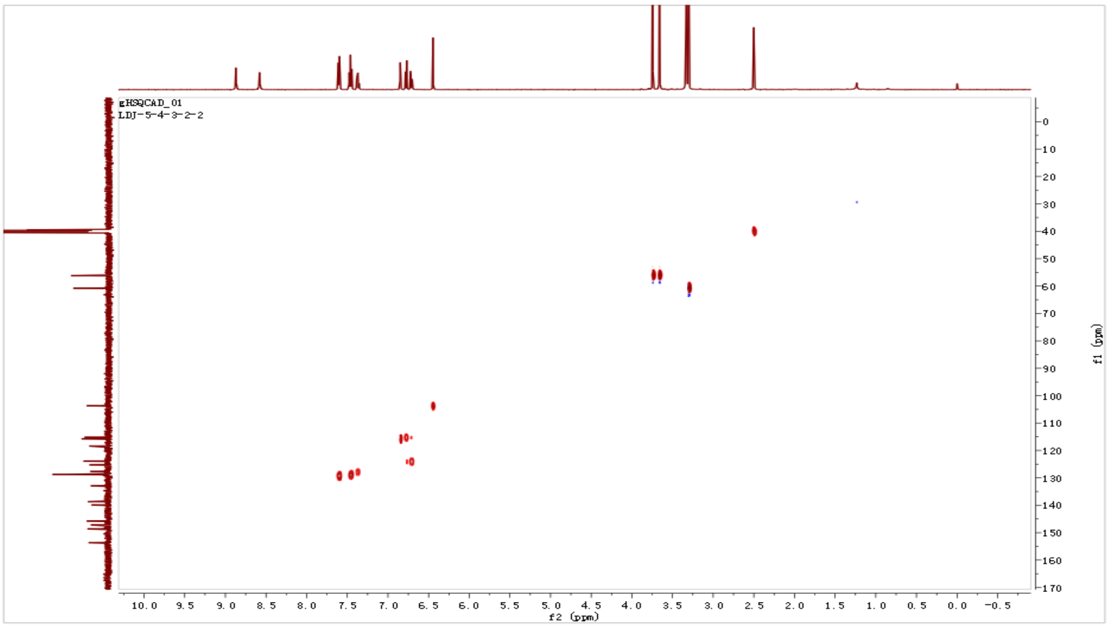

Figure S34. ^{1}H-^{1}H COSY spectrum of **5** in DMSO-d_6.

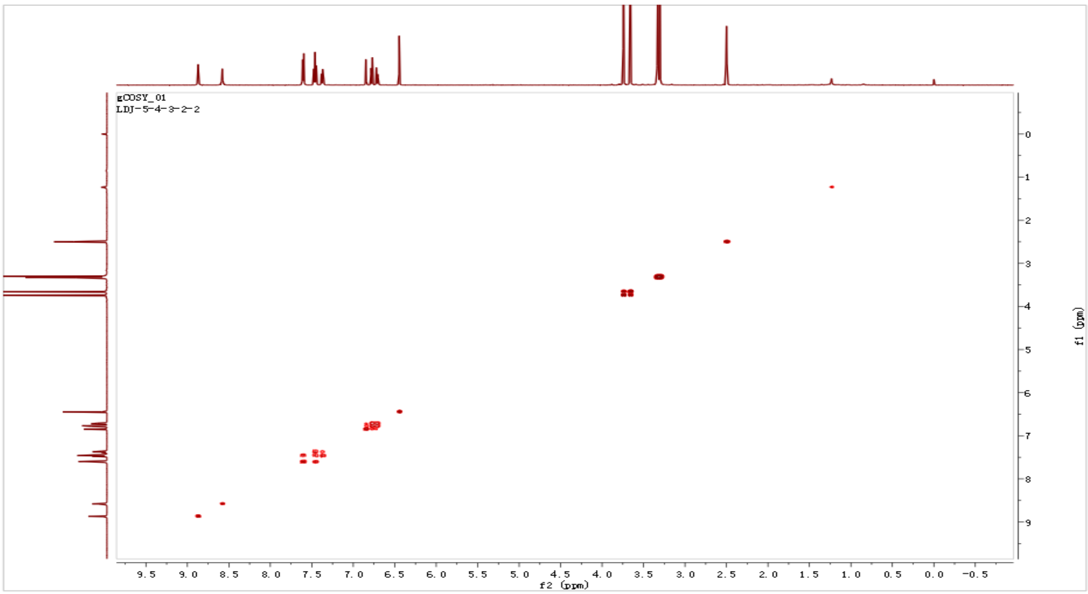

Figure S35. HMBC spectrum of **5** in DMSO-d_6.

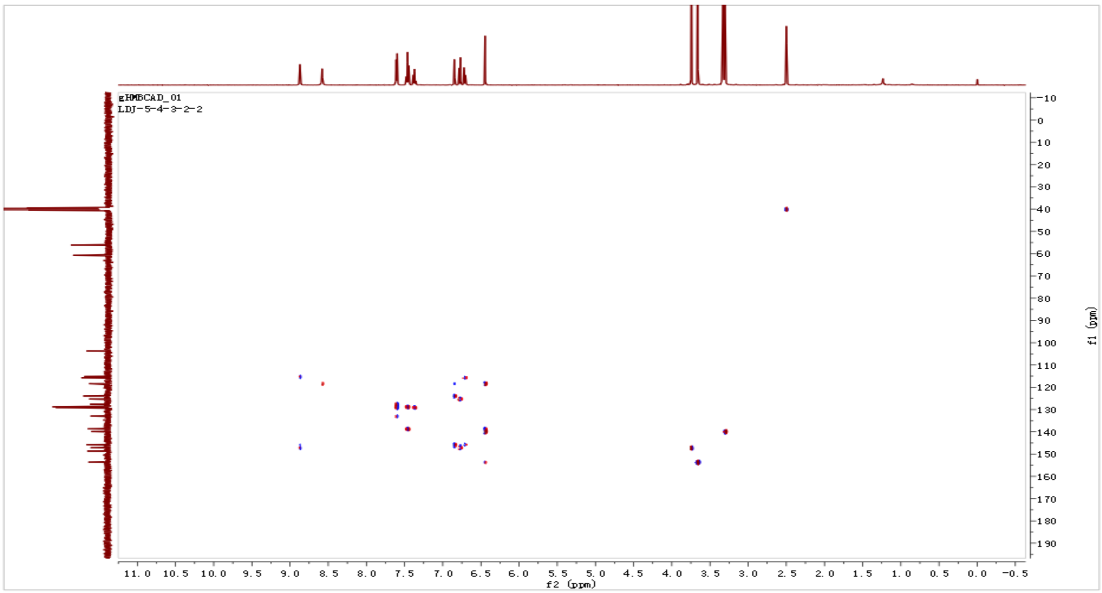

Figure S36. HRESIMS spectrum of **5**.

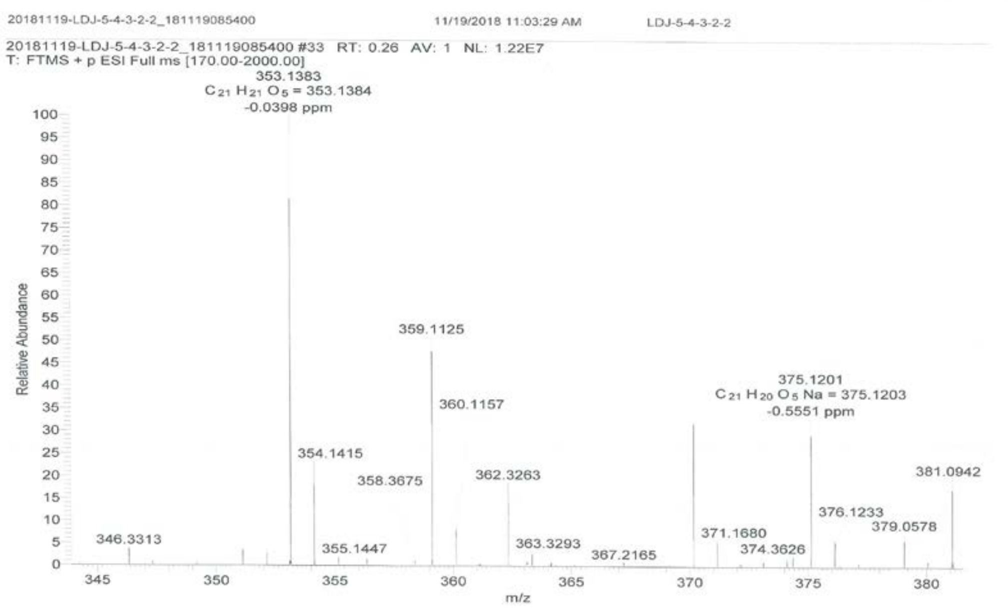

Figure S37. ^{1}H NMR (500 MHz, DMSO-d_6) spectrum of **6**.

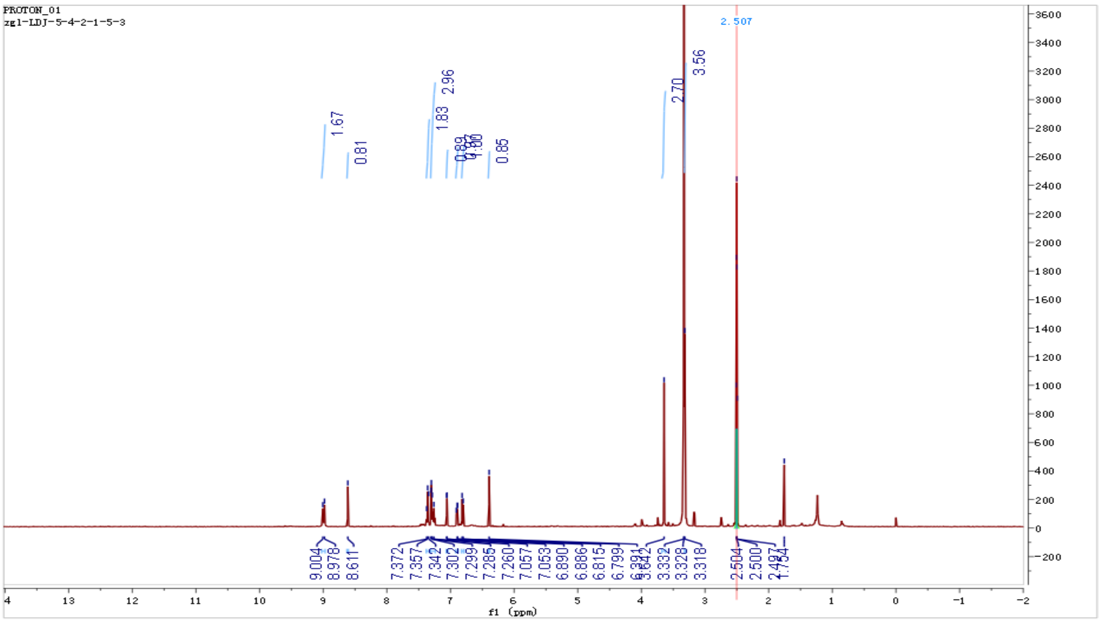

Figure S38. ^{13}C NMR (125 MHz, DMSO-d_6) spectrum of **6**.

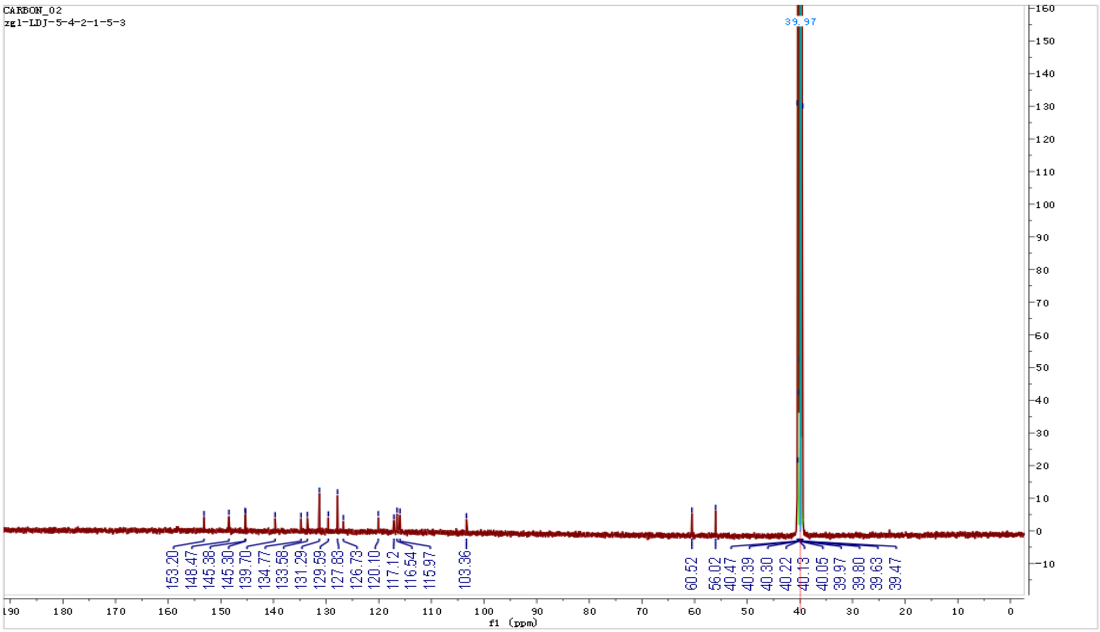

Figure S39. DEPT (125 MHz, DMSO-d_6) spectrum of **6**.

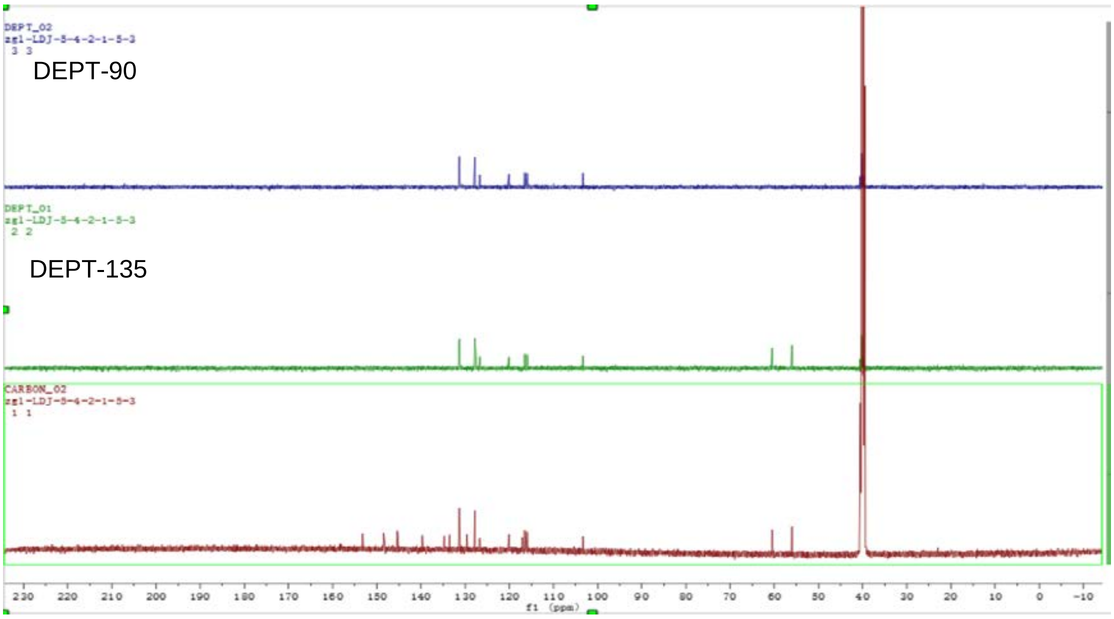

Figure S40. HSQC spectrum of **6** in DMSO-d_6.

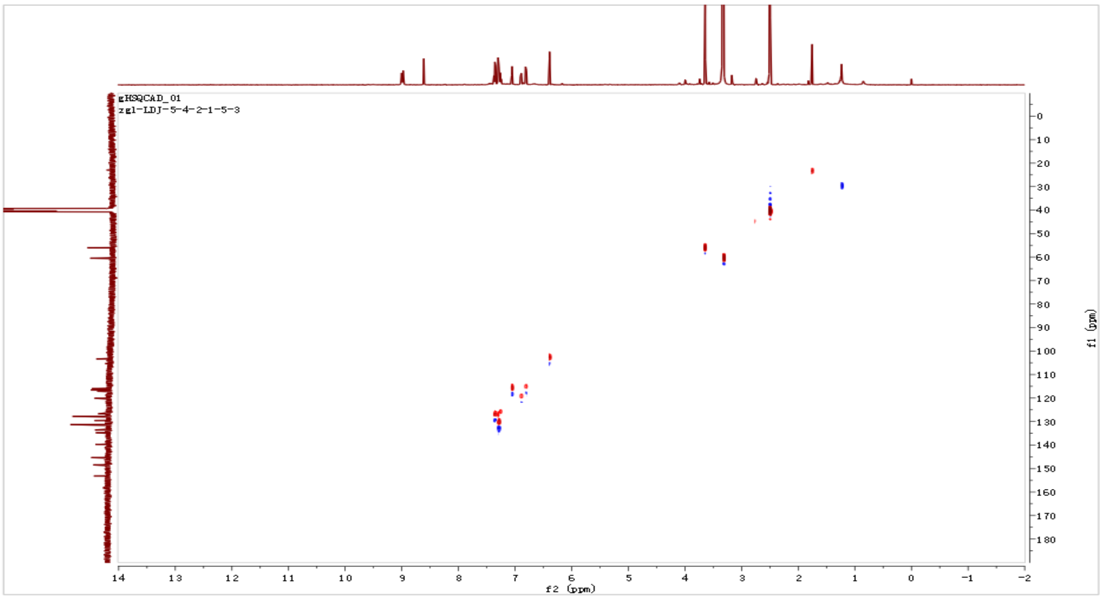

Figure S41. ^{1}H-^{1}H COSY spectrum of **6** in DMSO-d_6.

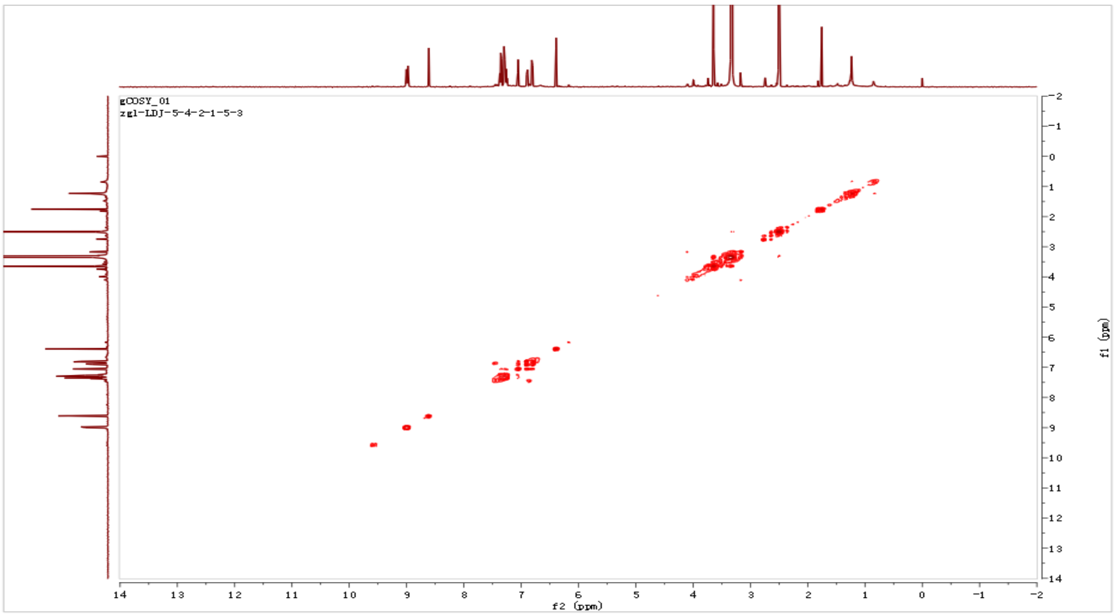

Figure S42. HMBC spectrum of **6** in DMSO-d_6.

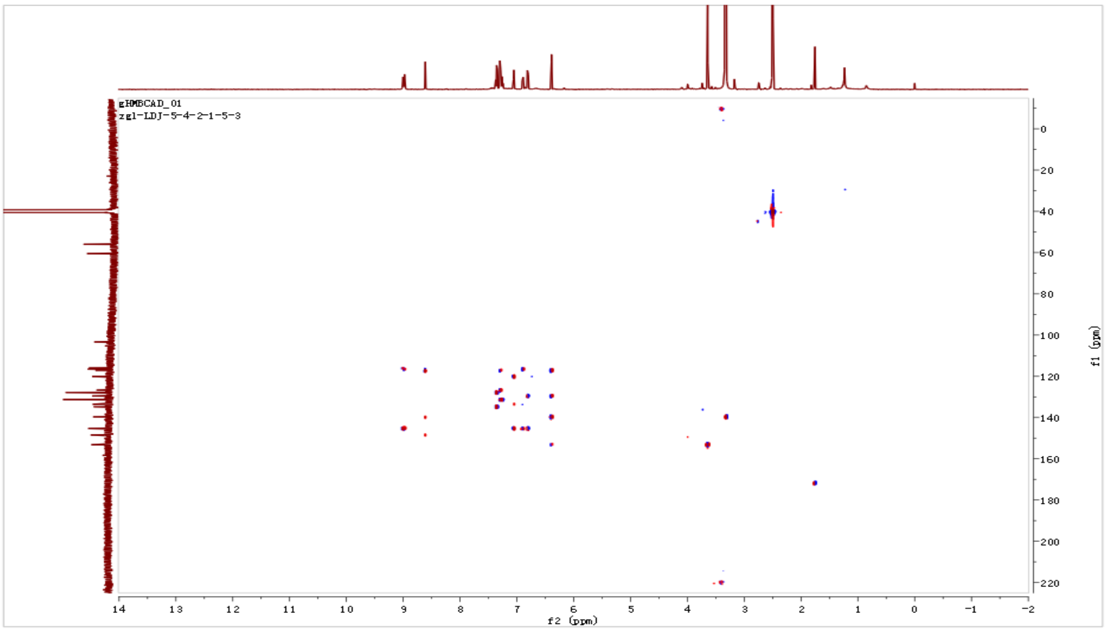

Figure S43. HRESIMS spectrum of **6**.

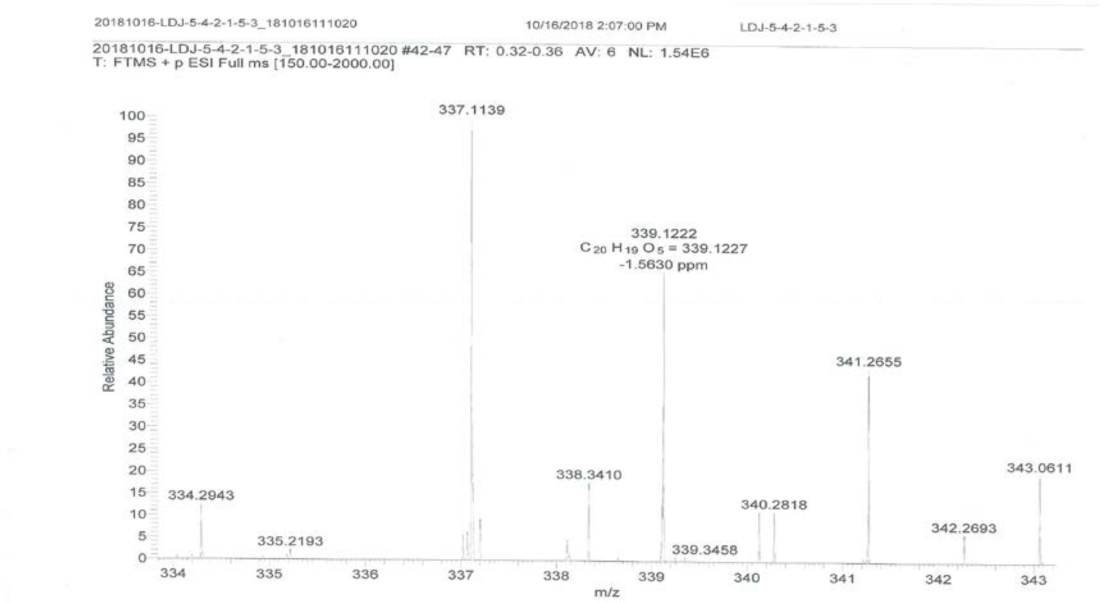

Figure S44. ^{1}H NMR (500 MHz, DMSO-d_6) spectrum of **7**.

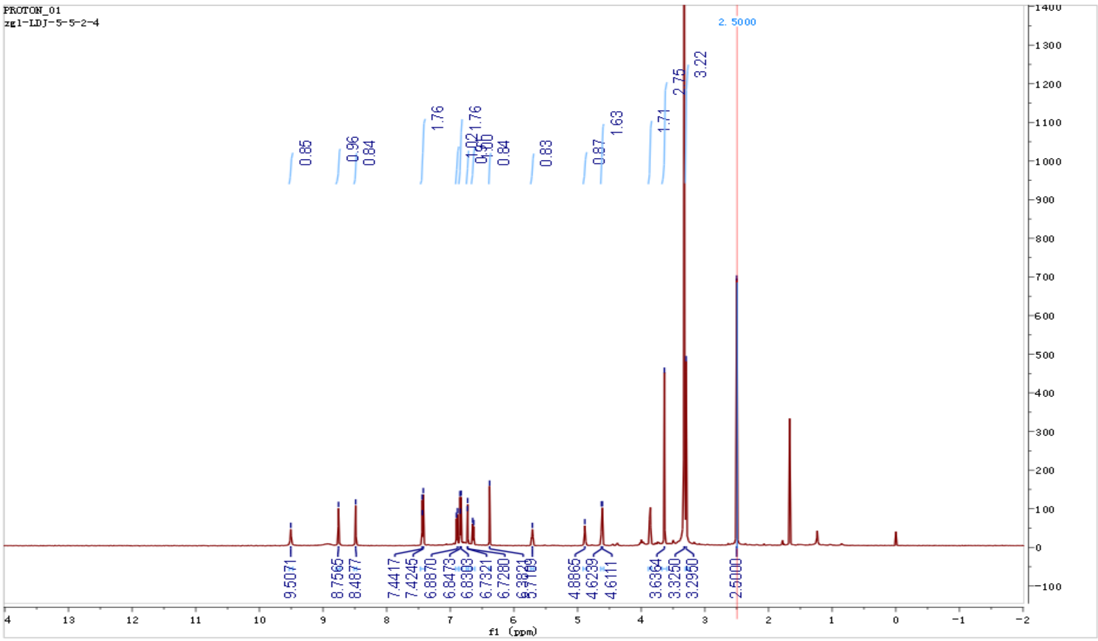

Figure S45. ^{13}C NMR (125 MHz, DMSO-d$_6$) spectrum of **7**.

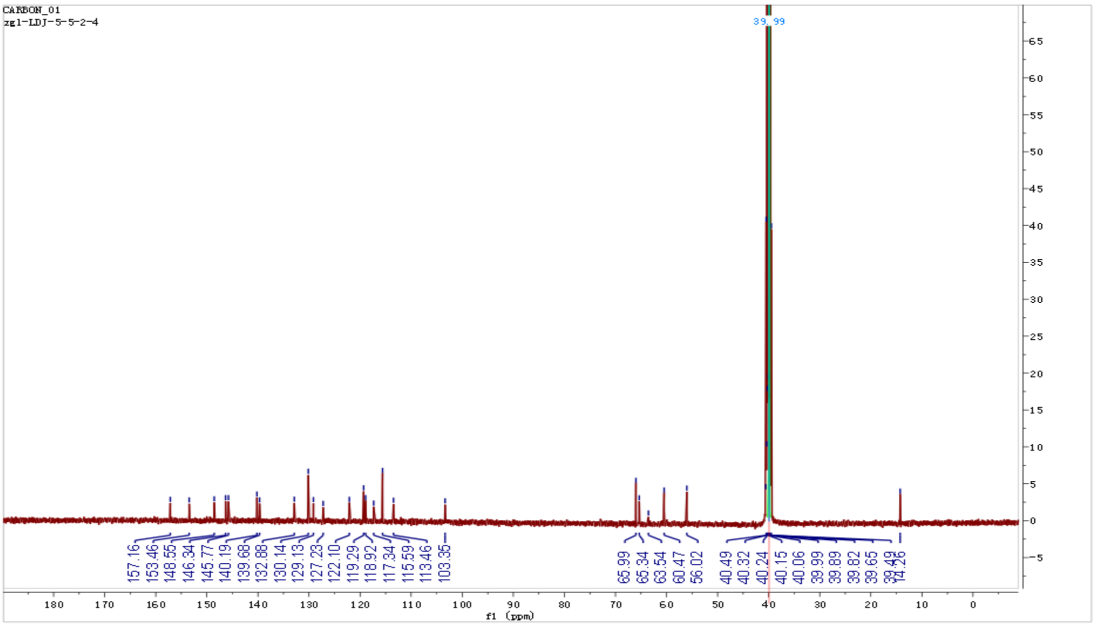

Figure S46. DEPT (125 MHz, DMSO-d$_6$) spectrum of **7**.

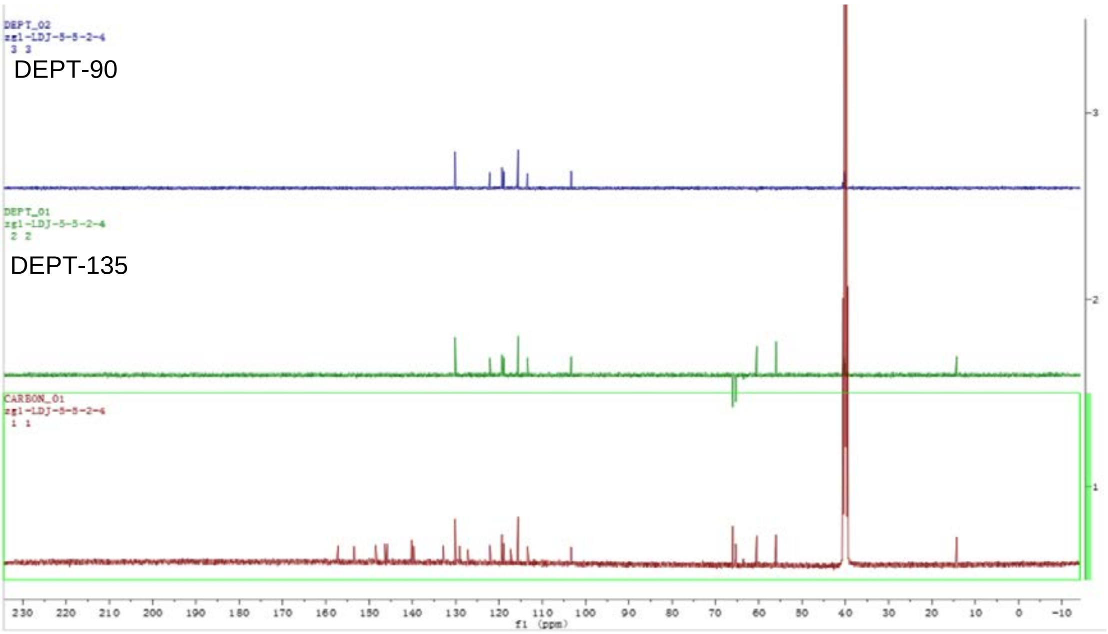

Figure S47. HSQC spectrum of **7** in DMSO-d_6.

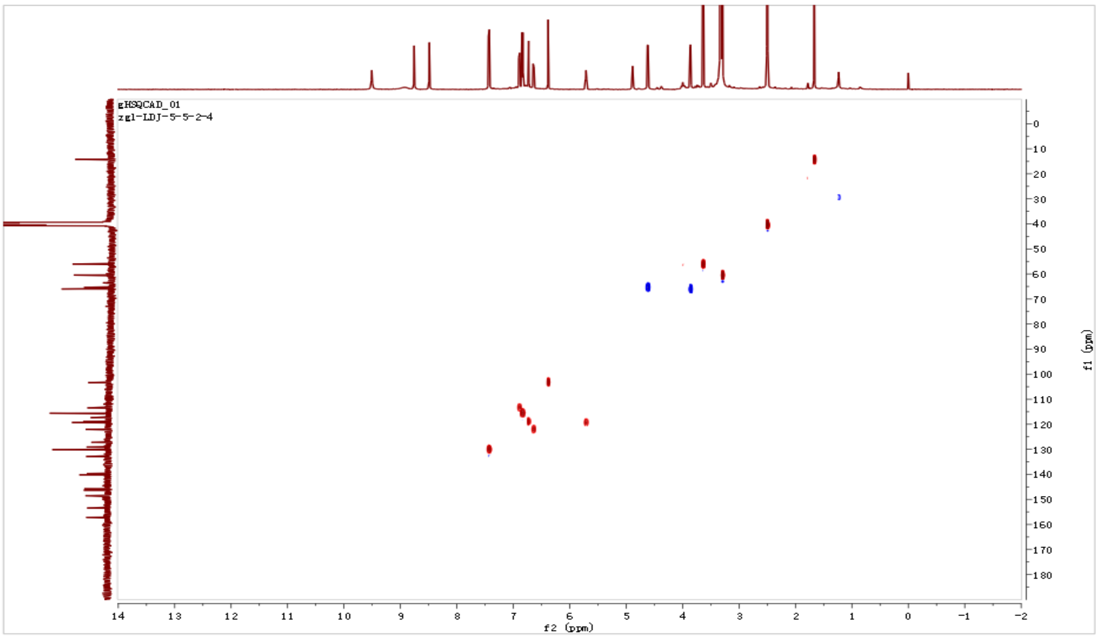

Figure S48. ^{1}H-^{1}H COSY spectrum of **7** in DMSO-d_6.

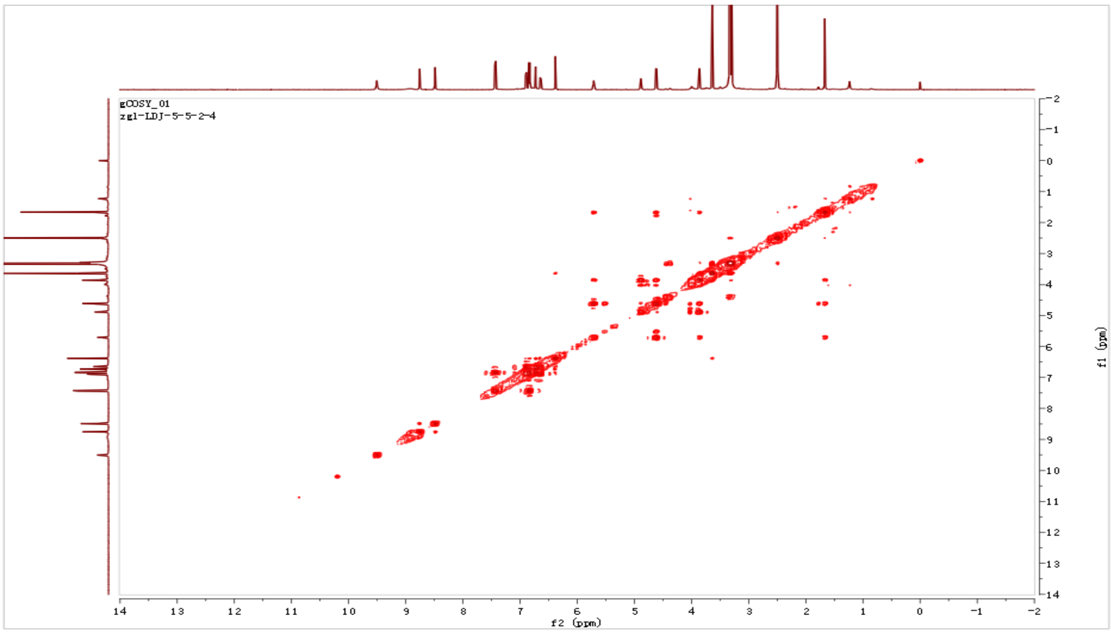

Figure S49. HMBC spectrum of **7** in DMSO-d_6.

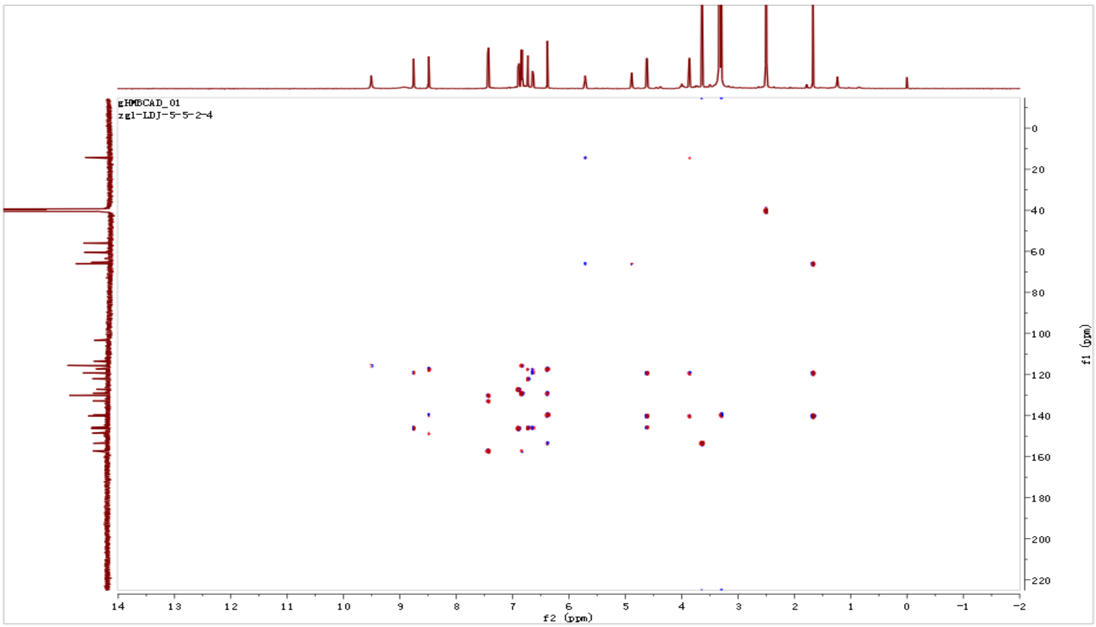

Figure S50. NOESY spectrum of **7** in DMSO-d_6.

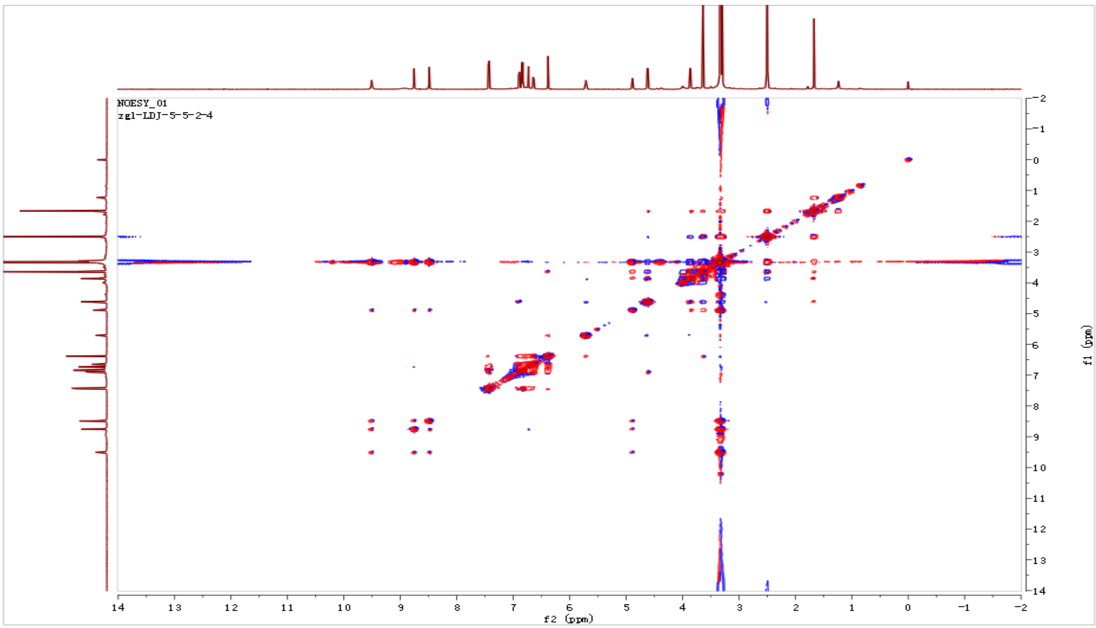

Figure S51. HRESIMS spectrum of **7**.

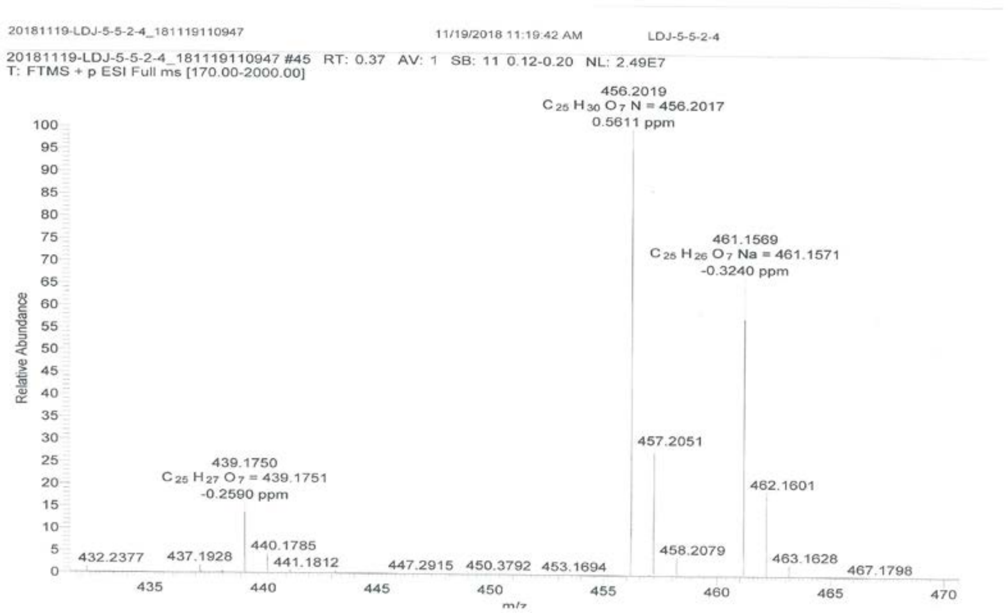

Figure S52. HPLC of LDJ-5 crude extract.

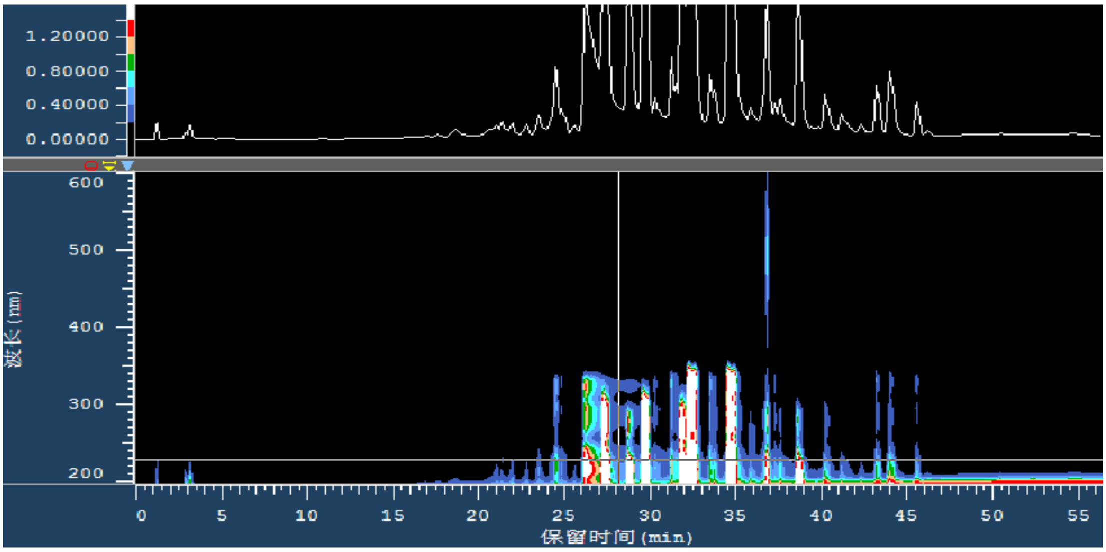

Figure S53. Chiral HPLC analysis of **1**.

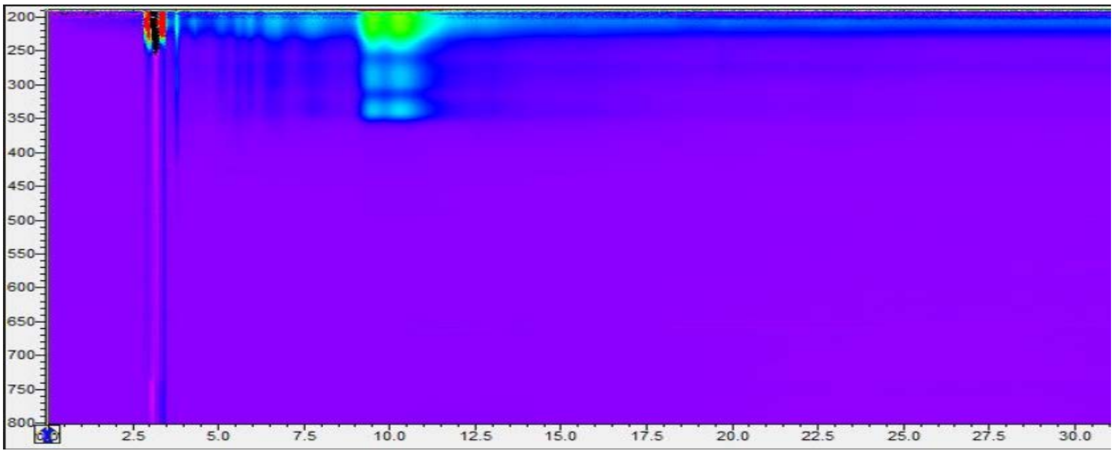

Figure S54. IR spectrum of **1**.

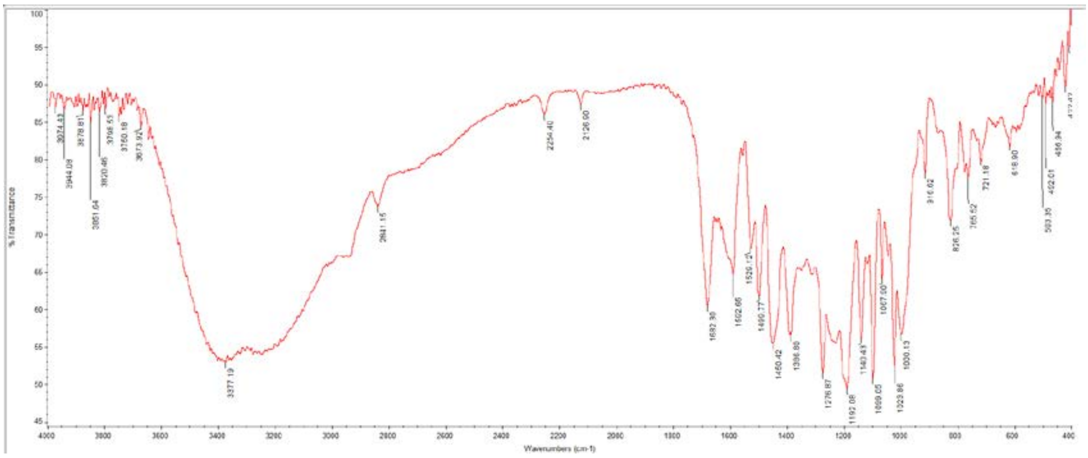

Figure S55. IR spectrum of **2**.

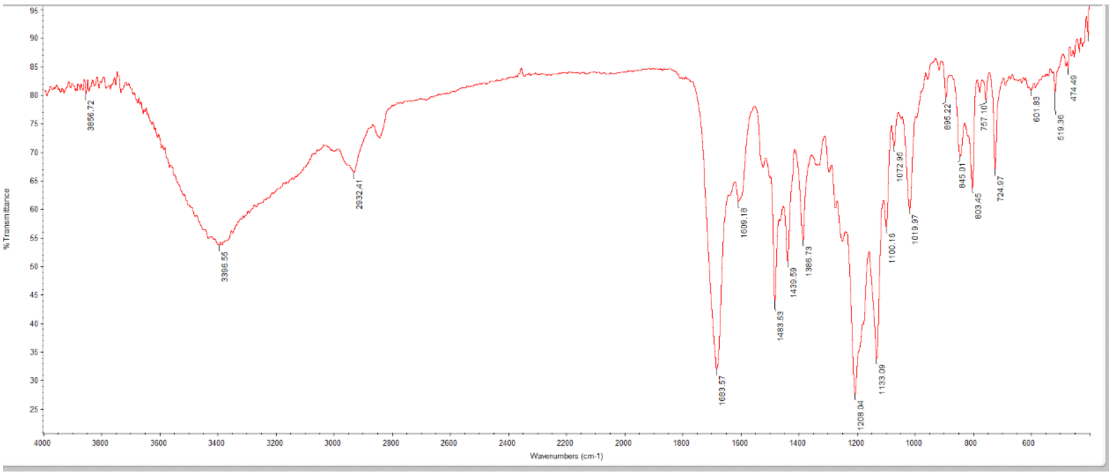

Figure S56. IR spectrum of **3**.

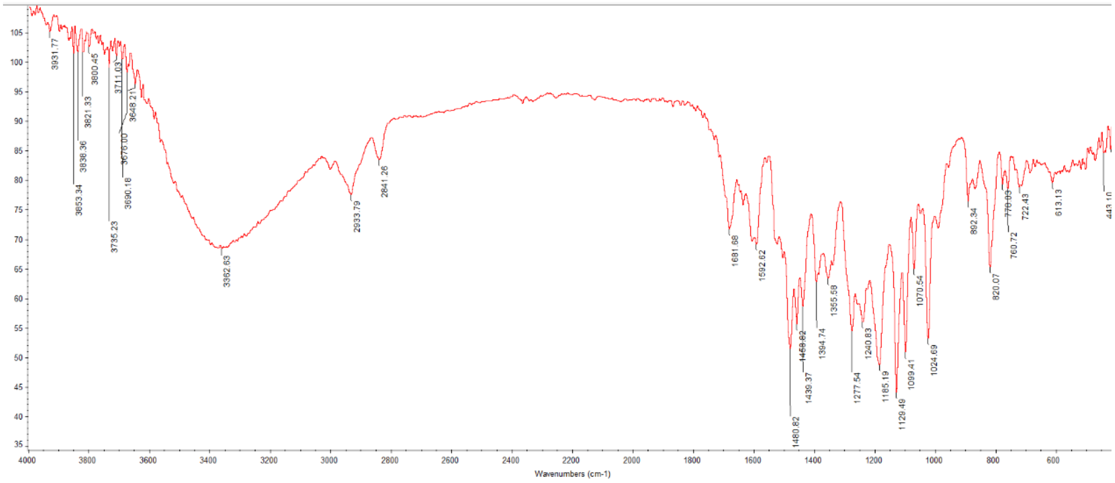

Figure S57. IR spectrum of **4**.

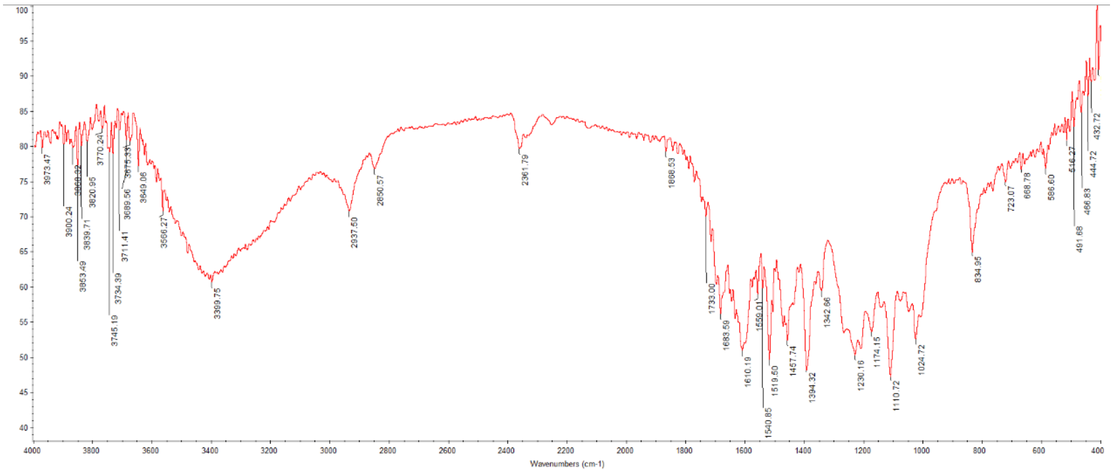

Figure S58. IR spectrum of **5**.

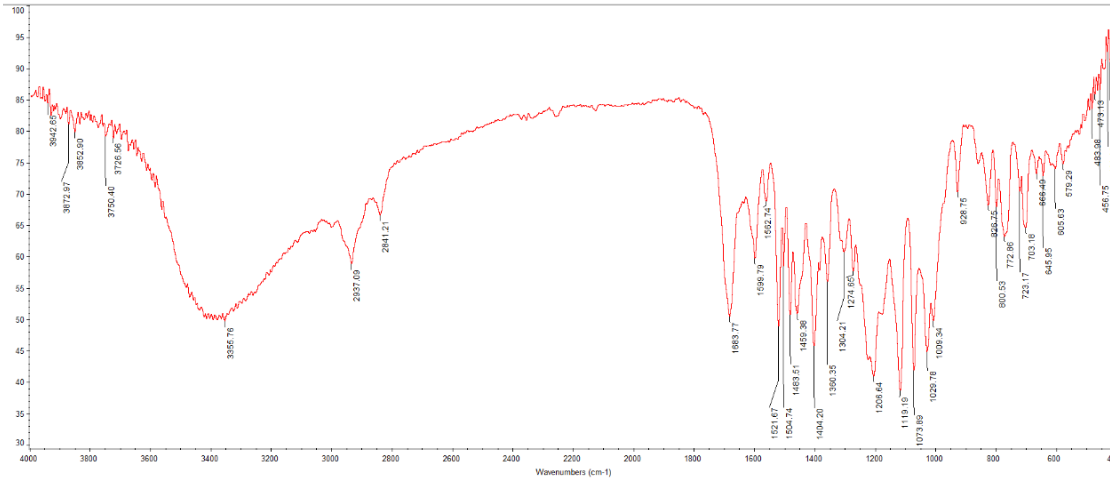

Figure S59. IR spectrum of **6**.

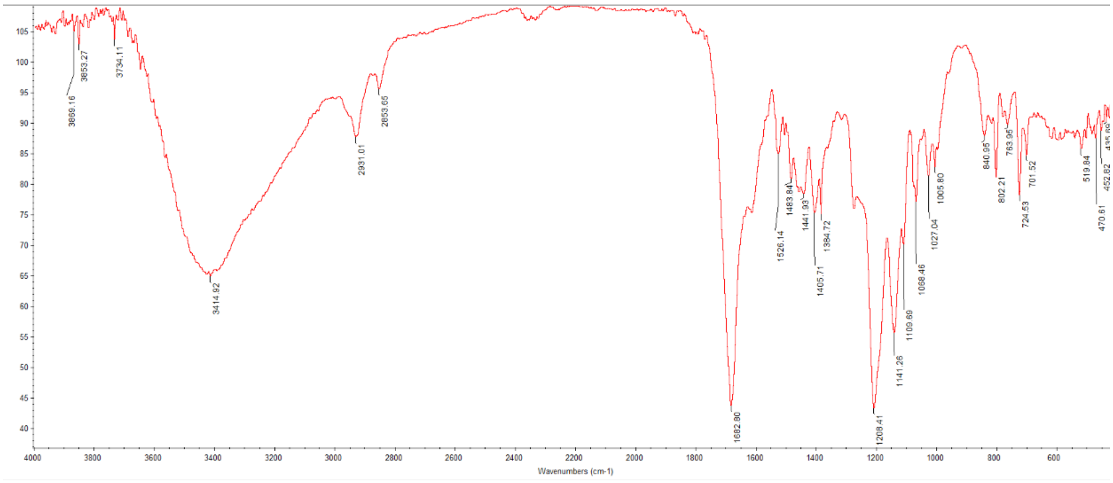

Figure S60. IR spectrum of **7**.

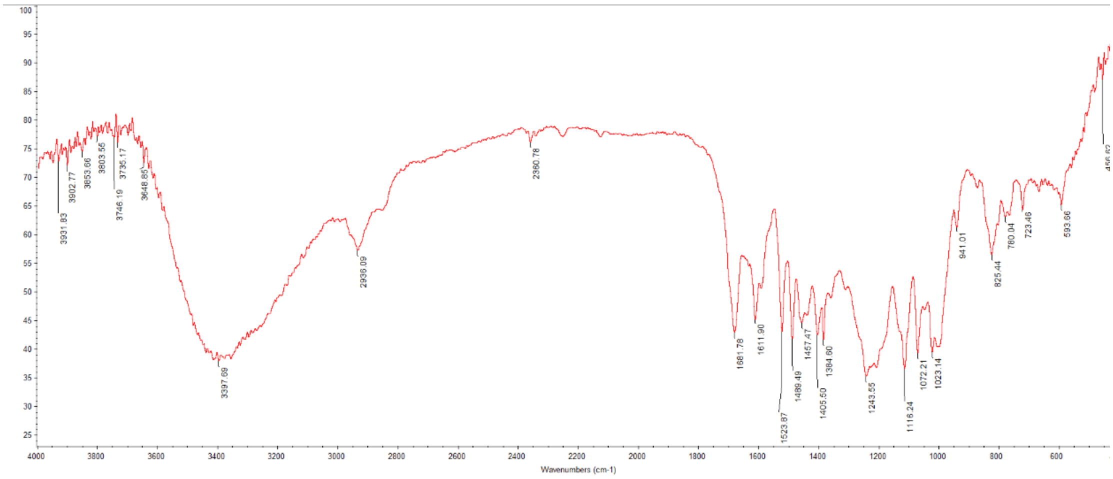

Table S1. Antimicrobial activity of **1-7**.

Compound	MIC (μg/mL)				
	Proteus species	*Pseudomonas aeruginosa*	*Bacillus subtilis*	*Bacillus cereus*	*Mycobacterium phlei*
**1**	>200	>200	>200	>200	>200
**2**	>200	40	>200	>200	79
**3**	19	>200	38	>200	38
**4**	>200	>200	>200	70	>200
**5**	35	70	70	70	70
**6**	>200	>200	>200	>200	>200
**7**	>200	>200	>200	87	>200
Positive drug[a]	0.26	0.52	4.14	2.07	0.52

[a]: Ciprofloxacin = positive control for *P.* species, *P. aeruginosa*, *B. subtilis*, *Bacillus cereus* and *Mycobacterium phlei*.
